# Effects of an Explicit Value Clarification Method With Computer-Tailored Advice on the Effectiveness of a Web-Based Smoking Cessation Decision Aid: Findings From a Randomized Controlled Trial

**DOI:** 10.2196/34246

**Published:** 2022-07-15

**Authors:** Thomas Gültzow, Eline Suzanne Smit, Rik Crutzen, Shahab Jolani, Ciska Hoving, Carmen D Dirksen

**Affiliations:** 1 Department of Health Promotion Care and Public Health Research Institute Maastricht University Maastricht Netherlands; 2 Department of Work & Social Psychology Faculty of Psychology and Neuroscience Maastricht University Maastricht Netherlands; 3 Department of Communication Science Amsterdam School of Communication Research University of Amsterdam Amsterdam Netherlands; 4 Department of Methodology and Statistics Care and Public Health Research Institute Maastricht University Maastricht Netherlands; 5 Department of Clinical Epidemiology and Medical Technology Assessment Care and Public Health Research Institute Maastricht University Medical Centre Maastricht Netherlands

**Keywords:** digital health, decision-making, decision support technique, decision aid, smoking, smoking cessation, informed decision-making, decision support, decision support tool, eHealth, evidence-based medicine, value clarification method

## Abstract

**Background:**

Smoking continues to be a driver of mortality. Various forms of evidence-based cessation assistance exist; however, their use is limited. The choice between them may also induce decisional conflict. Offering decision aids (DAs) may be beneficial; however, insights into their effective elements are lacking.

**Objective:**

This study tested the added value of an effective element (ie, an “explicit value clarification method” paired with computer-tailored advice indicating the most fitting cessation assistance) of a web-based smoking cessation DA.

**Methods:**

A web-based randomized controlled trial was conducted among smokers motivated to stop smoking within 6 months. The intervention group received a DA with the aforementioned elements, and the control group received the same DA without these elements. The primary outcome measure was 7-day point prevalence abstinence 6 months after baseline (time point 3 [t=3]). Secondary outcome measures were 7-day point prevalence of abstinence 1 month after baseline (time point 2 [t=2]), evidence-based cessation assistance use (t=2 and t=3), and decisional conflict (immediately after DA; time point 1). Logistic and linear regression analyses were performed to assess the outcomes. Analyses were conducted following 2 (decisional conflict) and 3 (smoking cessation) outcome scenarios: complete cases, worst-case scenario (assuming that dropouts still smoked), and multiple imputations. A priori sample size calculation indicated that 796 participants were needed. The participants were mainly recruited on the web (eg, social media). All the data were self-reported.

**Results:**

Overall, 2375 participants were randomized (intervention n=1164, 49.01%), of whom 599 (25.22%; intervention n=275, 45.91%) completed the DAs, and 276 (11.62%; intervention n=143, 51.81%), 97 (4.08%; intervention n=54, 55.67%), and 103 (4.34%; intervention n=56, 54.37%) completed time point 1, t=2, and t=3, respectively. More participants stopped smoking in the intervention group (23/63, 37%) than in the control group (14/52, 27%) after 6 months; however, this was only statistically significant in the worst-case scenario (crude P=.02; adjusted P=.04). Effects on the secondary outcomes were only observed for smoking abstinence after 1 month (15/55, 27%, compared with 7/46, 15%, in the crude and adjusted models, respectively; P=.02) and for cessation assistance uptake after 1 month (26/56, 46% compared with 18/47, 38% only in the crude model; P=.04) and 6 months (38/61, 62% compared with 26/50, 52%; crude P=.01; adjusted P=.02) but only in the worst-case scenario. Nonuse attrition was 34.19% higher in the intervention group than in the control group (P<.001).

**Conclusions:**

Currently, we cannot confidently recommend the inclusion of explicit value clarification methods and computer-tailored advice. However, they might result in higher nonuse attrition rates, thereby limiting their potential. As a lack of statistical power may have influenced the outcomes, we recommend replicating this study with some adaptations based on the lessons learned.

**Trial Registration:**

Netherlands Trial Register NL8270; https://www.trialregister.nl/trial/8270

**International Registered Report Identifier (IRRID):**

RR2-10.2196/21772

## Introduction

### Background

Smoking continues to be a major driver of global mortality [1], a trend that is mirrored in the Netherlands [2,3], which showcases that smoking prevention is imperative. However, as 21.7% of the adult Dutch population still smokes, according to recent figures [2], it is also of the utmost importance to invest in effective and evidence-based cessation assistance. Several cessation assistance tools are currently recognized as effective in aiding individuals in successfully achieving smoking abstinence. These range from behavioral interventions (eg, counseling [4]) to nicotine replacement therapy [5] and prescription medication [6]. Unfortunately, evidence-based cessation assistance is underused in various countries, including the Netherlands [7].

A common barrier to cessation assistance use is incorrect knowledge of the safety and efficacy of cessation assistance [8]. Therefore, increasing individuals’ knowledge may be a worthwhile avenue to explore when it comes to successfully increasing individuals’ cessation rates. In particular, being uninformed can lead to a state of uncertainty about which course of action to take—better known as decisional conflict [9]—which, in turn, is known to increase the chances of decision delay [9,10]. Therefore, individuals who decide to stop smoking but are uninformed about the possibilities of cessation assistance might delay the decision on *how* to stop smoking or simply decide to use the most common approach: attempting to quit smoking without the use of cessation assistance [11]. That said, knowledge provision alone is often insufficient to facilitate behavior change. This is commonly referred to as the knowledge-behavior gap in health promotion research [12,13]. Thus, knowledge provision alone might not be enough to support smokers motivated to quit smoking in their decisions about smoking cessation assistance. However, providing individuals who are motivated to stop smoking with decision support that also includes accurate information (next to other decision support elements) about cessation assistance tools might decrease decisional conflict. This, in turn, might facilitate smoking cessation efforts and ultimately increase the chances of long-term smoking abstinence.

Such decision support can be provided in the form of decision aids (DAs), which are interventions specifically designed to facilitate decisional processes [14]. A recent systematic review by Moyo et al [15] showed that DAs can be beneficial for smoking cessation, although traditionally, DAs have most often been applied to treatment and screening decisions rather than lifestyle-related decisions [14]. For example, BinDhim et al [16] showed that smoking cessation DAs can result in an increase in continuous abstinence at 1, 3, and 6 months compared with an intervention containing information only. Participants in the DA group were also more likely to have made an informed choice and showed fewer decisional conflict. However, information about the effective elements of smoking cessation DAs is currently lacking, and the only smoking cessation DA that has been previously studied in a Dutch context [17] has shown several limitations: it was largely paper based, thereby limiting widespread dissemination; it lacked an interactive design, although interactivity has been shown to positively influence factors such as information comprehensibility and attitudinal beliefs [18,19]; and it did not explicitly include methods of helping end users become aware of what is important to them personally (in the DA literature, this is often referred to as value clarification [20]), although this is regarded as an active DA element [21,22]. Moreover, interestingly, this DA had a positive effect on smoking cessation success but not on the uptake of cessation assistance [17]. Improving cessation assistance uptake might further increase the effectiveness of smoking cessation DAs, and overcoming the aforementioned limitations could play a promising role in achieving this.

To illustrate, given that a lack of knowledge is considered a barrier to cessation assistance use, adding interactive elements to a smoking cessation DA might be particularly helpful as interactive elements can improve information comprehensibility and positively influence individuals’ beliefs [18,19]. In addition to the use of interactive elements, explicitly devoting attention to smokers’ personal values could also positively influence the effects of smoking cessation DAs. The International Patient Decision Aid Standards Collaboration (IPDAS) regards these so-called value clarification methods (VCMs) as active DA elements [21,22]. VCMs can be divided into 2 different formats. *Explicit* VCMs refer to exercises that actively engage users in an activity to clarify what is important to them personally (eg, scoring certain statements), whereas *implicit* VCMs refer to the provision of static information that is specifically linked to the decision at hand. In other words, explicit VCMs include an element of interactivity that implicit VCMs lack. Recently, scholars have started to pay more attention to studying the added value of explicit (as opposed to implicit) VCMs. Previous studies have shown that explicit VCMs seem to be more effective than implicit VCMs in terms of decision-making processes [23], especially in the long run [24] and when people are supported in understanding the implications of their clarified values [25,26]. An approach to facilitate the understanding of the implications of clarified values is to show participants the options that best fit their clarified values [25]; for example, by providing computer-tailored advice based on answers provided in the explicit VCM. However, to date, it has not been studied whether the addition of explicit VCMs paired with such advice positively affects smoking cessation outcomes. To advance our understanding of the effectiveness of smoking cessation DAs and support more people in the Netherlands to quit smoking successfully, we developed a web-based smoking cessation DA (called *VISOR*) that includes interactivity and an explicit VCM paired with computer-tailored advice and studied its effects in a randomized controlled trial (RCT).

### The Smoking Cessation DA VISOR

In accordance with the IPDAS guidelines for DA development [27,28], *VISOR* was developed by a steering team (TG, ESS, CDD, and CH) that led a development process involving both professional experts and potential end users, for example, by assessing their needs and opinions before the initial development [29] and by conducting usability tests—this development process is described in detail elsewhere [28].

Moreover, we used the self-determination theory (SDT) [30] as the theoretical background. The SDT revolves around the formation of motivation and posits that 3 psychological needs (ie, the needs for autonomy, relatedness, and competence) are essential to developing autonomous motivation (ie, motivation that emanates from oneself and is not primarily externally motivated) [30,31]. Autonomous motivation is assumed to have a greater influence on long-term behavior change compared with more controlled forms of motivation [32]. The SDT is particularly well-suited to support DA developers because it focuses on perceived autonomy as opposed to theories that are often used to develop more persuasive interventions. DAs are similarly geared toward personal autonomy. Therefore, the SDT supported us in developing a DA that not only provided accurate information but also helped motivate end users, for example, by framing the information in the DA autonomy supportively. Finally, *VISOR* took a stepwise approach to facilitate autonomous decision-making, which is concordant with personal values and smoking behavior. *VISOR* comprised 8 sections that are described in Textbox 1*.*

**Figure 1:**
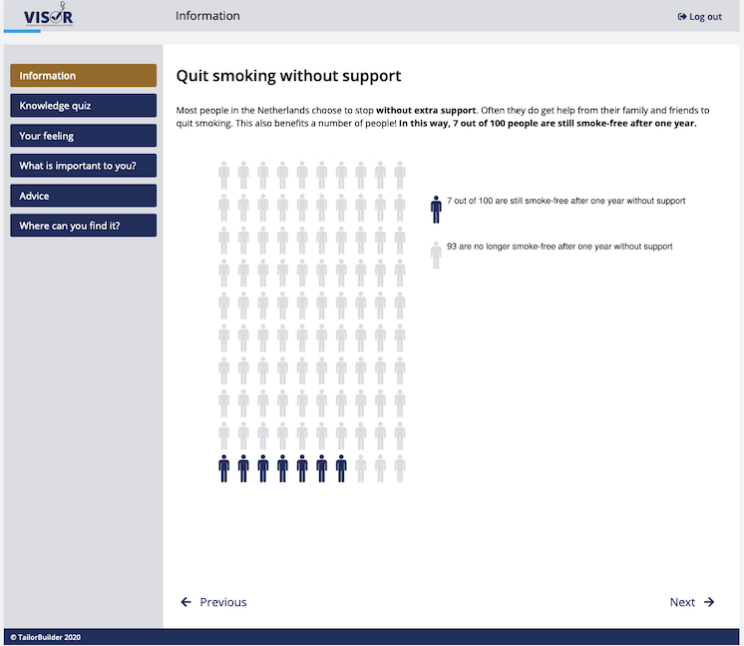
Screenshot of the information section in the decision aid (original text translated from Dutch); the displayed icon array has been created using IconArray [33].

**Figure 2:**
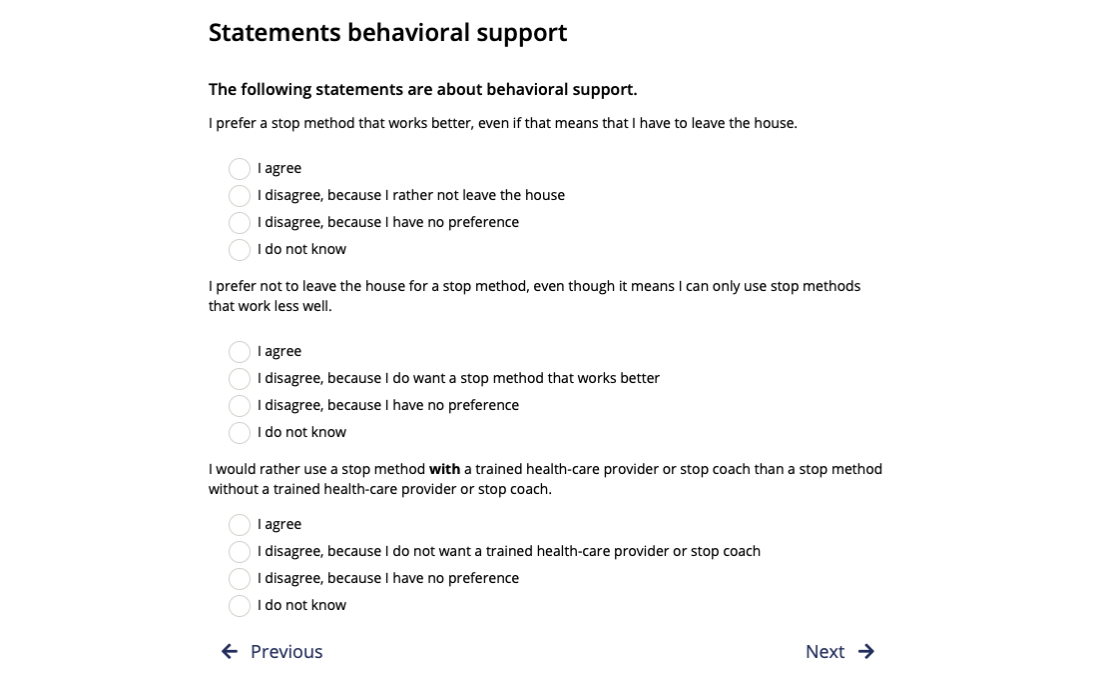
Screenshot of a part of the VCM in the decision aid (original text translated from Dutch). VCM: value clarification method.

*VISOR* was a stand-alone, 1-time intervention meant to support adult smokers in the general population in their decision to use smoking cessation assistance and did not have to be used together with a health care professional. That said, *VISOR* could be used to prepare patients and clients for a health care consultation about smoking (cessation), and certain cessation assistance options (eg, prescription medication) required a health care provider to prescribe them. Therefore, if *VISOR* users chose to use a cessation assistance option that required a prescription from a health care provider, they were advised to contact their health care provider to gain access to this specific option. More in-depth information on *VISOR*, including the specific theoretical underpinnings of each step, can be found elsewhere [28]. Examples of the information section and explicit VCMs are shown in Figure 1 (information section [33]) and Figure 2 (explicit VCMs). The screenshots were translated from the original Dutch to English to facilitate understanding.

**Overview of VISOR’s sections.**

**Overview of VISOR’s sections**

1. Information section explaining the decision at hand, as well as all the cessation assistance available in the Netherlands
2. Optional knowledge quiz
3. Brief smoking assessment
4. Intuitive decision between different clusters of cessation assistance tools:
  - Behavioral support
  - Nicotine replacement therapy (NRT)
  - Combination of behavioral support, NRT, and prescription medication
  - Combination of behavioral support and NRT
  - Combination of behavioral support and prescription medication
  - Other (non–evidence-based) cessation assistance
  - No cessation assistance at all
5. Intermediate advice to use a combination of behavioral and pharmacological cessation assistance tools, for users who chose the following:
  - Behavioral support only, or NRT only, while also indicating that they smoke >10 cigarettes on a normal day and/or have made ≥1 smoking cessation attempt or attempts in the past (in step 3)
  - Non–evidence-based cessation assistance or no cessation assistance at all in step 4 regardless of their answers in step 3
6. Explicit value clarification method (VCM) for users who chose evidence-based cessation assistance tools in steps 4 or 5, where users were asked to rate certain statements regarding cessation assistance characteristics (eg, “I prefer a stop method that works better, even if that means that I have to leave the house”).
  - Users only rated statements for options that belonged to the cluster of cessation assistance options they selected in the previous step or steps
7. Computer-tailored advice based on the explicit VCM, including an optional ranking of all options; only when it was possible to give clear advice, (ie, if users’ scores did not suggest that >2 cessation assistance tools were equally suitable based on their indicated values)
8. Access information on how to obtain the chosen cessation assistance (eg, nicotine patches)

### Study Goals and Hypotheses

In this study, the behavioral (ie, smoking abstinence and cessation assistance use) and decisional effects (ie, decisional conflict) of an explicit VCM paired with computer-tailored advice (within a smoking cessation DA) are reported. An RCT was conducted to investigate these effects. Specifically, we tested the following hypotheses (as described in the study protocol [28] and the Netherlands Trial Register [NL8270]):

- Hypothesis 1a or 1b is that a DA with explicit VCM and computer-tailored advice will lead to a statistically significant increase in smoking abstinence after 1 month (hypothesis 1a) and 6 months (hypothesis 1b) compared with a DA without explicit VCM and computer-tailored advice.
- Hypothesis 2a or 2b is that a DA with explicit VCM and computer-tailored advice will lead to a statistically significant increase in evidence-based cessation assistance use after 1 month (hypothesis 2a) and 6 months (hypothesis 2b) compared with a DA without explicit VCM and computer-tailored advice.
- Hypothesis 3 is that a DA with explicit VCM and computer-tailored advice will lead to a statistically significant decrease in decisional conflict (state of uncertainty about which course of action to take) immediately after using the DA compared with a DA without an explicit VCM and computer-tailored advice.

## Methods

### Overview

An RCT was conducted in line with the CONSORT-EHEALTH (Consolidated Standards of Reporting Trials of Electronic and Mobile Health Applications and Online Telehealth) checklist [34]. However, we deviated from the checklist in one aspect: participants were only invited for follow-up measurements if they completed one of the DAs; that is, if they completed the intervention until the end. This was because we wanted to ensure that the participants we included in the analysis received additional intervention elements as those were the focus of our RCT.

### Ethics Approval

The study did not fall under the scope of the Medical Research Involving Human Subjects Act, as indicated by the Medical Ethics Committee of Zuyderland, the Netherlands (16-N-227; International Registered Report Identifier: RR2-10.2196/21772), and the development of *VISOR* and the accompanying studies (eg, the aforementioned needs assessment [29]) were funded by the Dutch Cancer Society (UM2015-7744). Study materials (including the underlying data and statistical scripts) can be found on the open science framework (OSF) website [35].

### Primary and Secondary Outcomes

The primary outcome of this study was 7-day point prevalence after 6 months (corresponding to hypothesis 1b). Secondary outcomes were 7-day point prevalence after 1 month (corresponding to hypothesis 1a), evidence-based cessation assistance use after 1 and 6 months (corresponding to hypothesis 2a or 2b), and decisional conflict directly after using the DA (corresponding to hypothesis 3).

### Sample Size

An a priori power calculation was conducted based on the primary outcome measure of 7-day point prevalence abstinence and the only other RCT in the Netherlands, which tested the effect of a smoking cessation DA in which a significant effect (20.2% vs 13.6%) was found at 6 months [17]. To be able to significantly (α=.05; β=.20) detect the same effect in a 1-sided test, 398 smokers per arm were necessary at the end of the trial (796 in total). Considering 50% attrition over the study period, we aimed to include 1592 smokers at baseline.

### Study Population

Participants were included if they were (1) currently smoking, (2) motivated to stop smoking within 6 months, (3) aged between 18 and 100 years, (4) able to understand Dutch, and (5) had access to the internet and the necessary internet literacy (skills) to use the DA. The last 2 inclusion criteria were not actively screened but were deemed inherent to participation. Participants were excluded if they did not meet the inclusion criteria or exclusively used e-cigarettes. As described in the study protocol [28], participants were mainly recruited on the web to reflect the web-based nature of *VISOR*, and the entire trial was web-based (ie, there were no offline contacts). Recruitment took place mainly by using paid social media advertisements and unpaid social media posts on project accounts, [36] which were also shared on the team members’ accounts, their respective institutions, and other relevant organizations within the Netherlands. In addition, *VISOR* was featured in regional media (eg, a newspaper interview), and we used a project website with a direct access point to *VISOR* via a clickable button. Owing to the small influx of participants (especially after the start of the COVID-19 pandemic), we decided to deploy other additional recruitment activities as well, such as a study call, information in a relevant Dutch journal for general practitioners [37], and student pools at the Universities of Amsterdam and Maastricht. All (potential) participants received information about the content of *VISOR*, the duration of the study (including the number of follow-up measurements), and the compensation that they received for completing the last measurement (€10 [US $12.17]). Throughout the study, participants also received information on the duration of *VISOR* and the questionnaires. The participants received no information regarding the differences between the intervention and control groups to avoid bias in the results of the trial. The students at the University of Amsterdam received research credits instead of monetary compensation. All recruitment materials (eg, the project website) included a display of the project team’s institutional affiliations in some form.

### Intervention and Comparator Groups

Participants in the intervention group received the DA as described in the *Introduction* section (see *The Smoking Cessation DA VISOR* section), whereas participants in the control group received the same DA, excluding the explicit VCM and computer-tailored advice; that is, steps 6 and 7 described in Textbox 1 were skipped. The only other (small) difference was that participants in the intervention group were immediately directed toward the end after they had chosen to not use evidence-based cessation assistance (ie, step 8—access information—was skipped), which was not the case for the control group. Thus, both groups had a chance to re-evaluate their choice, as the intervention group was offered a chance to re-evaluate their choice during the additional elements. Neither the DA received by the intervention group nor that received by the control group changed throughout the trial. Additional information can be found in the study protocol [28].

### Trial Flow and Measurement Instruments

#### Overview

In total, the study comprised 4 fully automated and web-based contact moments: time point 0 for the baseline questionnaire and *VISOR*, time point 1 (t=1) directly after participants had used *VISOR*, time point 2 (t=2) after 1 month, and time point 3 (t=3) after 6 months. Participants were asked to fill in each follow-up questionnaire if they made use of the entire DA, even if they did not fill in one of the other follow-up questionnaires. To avoid high attrition rates, participants received either 1 automatic reminder after a week (if they had not filled in a follow-up questionnaire at all) or 2 after 2 days and a week (if they had already started filling in at least part of a follow-up questionnaire). Participants who started using *VISOR* or started filling in the baseline questionnaire (time point 0) without finishing it also received 2 automatic reminders (after 2 days and a week). In the last reminder for t=3, participants were also offered the option to share only their answers regarding the primary outcome (ie, 7-day point prevalence abstinence) with the research team. All data were self-assessed. If available, we used previously validated measurements [9] and measurements that were previously used in a Dutch context [38]; if possible, we used measurements that were previously used in self-administered web-based studies [39,40]. For more information, refer to the Checklist for Reporting Results of Internet E-Surveys checklist in Multimedia Appendix 1. In the case of psychological constructs collected using multiple items (eg, decisional conflict), we assessed scale quality, as proposed by Crutzen and Peters [41], using the *Rosetta Stats* package in R (R Foundation for Statistical Computing) [42,43] in two steps: (1) investigating scale structure by exploratory factor analysis and (2) calculating omega (Ω) [44] as a less biased alternative to (Cronbach) α.

Participants were registered for the study via a web-based form, which included their provision of informed consent and the creation of an account. Before account creation, participants were automatically randomized into either the intervention or the control group by the web-based platform on which questionnaires and *VISOR* were hosted, allocating approximately 50% of the respondents to either group. The end users were blinded to their allocated groups. Immediately after registration, the participants were asked to complete the baseline questionnaire. A visual representation of the trial flow can be found in the study protocol [28].

#### Baseline Measurements: Directly Before the DA

Demographic information was collected based on 3 criteria: age, gender, and education. Smoking behavior was collected regarding the used tobacco products, amount of tobacco consumption per product per day, past cessation attempts, amount of past cessation attempts (for people who previously attempted to stop smoking), and cessation assistance use in the past 6 months. If the participants indicated that they had used cessation assistance, they were also asked what had been used. Additional information regarding demographic information and smoking behavior can be found in Multimedia Appendix 2 [45-47].

Nicotine dependence was measured using the Revised Fagerström Test for Nicotine Dependence (FTND-R), which has shown better psychometric properties than the unrevised version [48] and was verified to be unidimensional within our sample (Ω=0.76; further information can be found on the OSF [35]). We changed the wording of the items slightly (eg, *smoking moment* instead of *cigarette*) to include other tobacco products as well and left out the item relating to the amount of cigarette consumption. Rather, we created a composite score based on participants’ answers to the item about the number of tobacco products, expressed as the number of cigarettes, with 1 hand-rolled cigarette and 1 other or a cannabis product equaling 1 cigarette, 1 pipe equaling 2.5 cigarettes, and 1 cigar equaling 4 cigarettes [38,49]. Subsequently, this composite score was recoded in line with the FTND-R (0=0-10 cigarettes, 1=11-20 cigarettes, 2=21-30 cigarettes, and 3=more than 30 cigarettes). We had to exclude e-cigarette use for this composite score as it was unclear how the answer categories of the e-cigarette use item related to the amount of cigarette consumption. Subsequently, a composite score was created by summing this item with the 5 other FTND-R items, resulting in 1 FTND-R score per individual ranging from 0 to 16, with 0 indicating no dependence.

Finally, the stage of decision-making was measured with 1 item: “Have you thought about how to quit smoking at this point? Choose the answer that best suits your situation; 1=I haven’t begun to think about the choices, 2=I haven’t begun to think about the choices, but am interested in doing so, 3=I am considering the options now, 4=I am close to selecting an option, 5=I have already made a decision, but am still willing to reconsider, 6=I have already made a decision and am unlikely to change my mind” [50].

#### Follow-up at t=1: Directly After the DA

After the DA, we measured decisional conflict (secondary outcome) using the Decisional Conflict Scale [9], which was also verified to be unidimensional in our sample (Ω=0.98; further information can be found on the OSF [35]). We used all 16 items using the original statement format with 5 response categories (0=strongly agree, 1=agree, 2=neither agree nor disagree, 3=disagree, and 4=strongly disagree) and created a composite score as described in the user manual provided by O’Connor [51]: individuals’ scores were summed, divided by 16, and multiplied by 25. Thus, every participant had a score ranging from 0 (no decisional conflict) to 100 (extremely high decisional conflict). Scores >37.5 were generally regarded as being associated with decision delay or being unsure about decision implementation [51].

#### Follow-ups at t=2 and t=3: 1 Month and 6 Months After Baseline

After 1 month and 6 months, participants were queried regarding the choice they made (ie, the implemented decision; secondary outcome) and whether they were able to abstain from smoking in the previous 7 days (ie, 7-day point prevalence abstinence; secondary and primary outcomes).

### Data Analysis

All analyses were performed in R [43] using the integrated development environment of RStudio [52]. First, descriptive analyses were conducted to assess the sample characteristics.

Second, to determine which factors influenced nonuse attrition (ie, attrition during the intervention) and dropout attrition (ie, not returning to the follow-ups) [53], we first compared participants at baseline who did not finish *VISOR* with those who finished *VISOR* using the Wilcoxon rank-sum test, Mood median test, chi-square test, or Fisher exact test, depending on the variable in question. Subsequently, we checked the results of these univariate analyses by using logistic regression with nonuse attrition as the outcome and all significant variables from the univariate analyses as predictors. Regarding dropout attrition, we compared participants at t=1, t=2, and t=3 with those lost to follow-up (after having used the intervention) using the same approach as described previously.

Third, logistic regression was used to test hypotheses 1a, 1b, 2a, and 2b. First, we conducted crude analyses in which we only included the allocated group (ie, the intervention group compared with the control group) as a predictor and 7-day point prevalence abstinence as the outcome, followed by fully adjusted analyses in which we corrected for age, gender, education, and the FTND-R [28,54]. All covariates were selected a priori [28,54,55]; however, because of multicollinearity issues in some of the adjusted models testing hypotheses 2a and 2b, we needed to recode the educational variable into low and high rather than low, medium, and high (as for the other analyses). This was only done for the models where this was the case. In addition, because of the size of the 2 very small gender (identity) groups (ie, nonbinary participants and participants who preferred not to state their gender), we were unable to include participants belonging to these groups in all adjusted models. Therefore, we decided to include the data of those participants in the crude models but not in the adjusted models. To test the robustness of the results, logistic regression was conducted according to three different approaches: (1) complete cases only, (2) worst-case scenario (dropout respondents were considered to still smoke; ie, penalized imputation), and (3) multiple imputations (MIs) using the *mice* package [56]. Variables that were included in the imputation model can be found in Multimedia Appendix 3*.* In the MI models, we accounted for a technical mistake described in the *Changes From the Study Protocol* section. As we had directional hypotheses, we conducted 1-sided tests as planned a priori [28]. To calculate the P values, we used the following formulas: (1) *P*/2 if the effect moved in the hypothesized direction and (2) 1 – (*P*/2) if the effect moved in the other direction. Similarly, we swapped the upper bound of the calculated CIs to infinity. This was only done for the intervention effect as we had no specific directional hypotheses regarding the covariates.

Finally, hypothesis 3 was tested using linear regression. Again, we started with the crude analyses, using the allocated group as a predictor and the Decisional Conflict Scale as the outcome, followed by fully adjusted analyses corrected for covariates selected a priori (ie, age, gender, education, and stage of decision-making) [28,54,55]. Again, we had to exclude participants from certain analyses because they belonged to a very small gender (identity) group. Regarding hypothesis 3, we used only (1) complete cases and (2) MI using the *mice* package [56]. Variables that were included in the imputation model can be found in Multimedia Appendix 4*.* Again, we calculated the values for the 1-sided tests as described previously; however, as we expected a negative effect, we swapped the lower bound of the calculated CIs with infinity. In complete case analyses, all participants who filled in the respective outcome measures (eg, everyone who filled in the entire Decisional Conflict Scale) were included, even when participants did not finish the entire follow-up questionnaire.

### Changes From the Study Protocol

In the original study protocol, and as described in the Netherlands Trial Register, participants were contacted 4 times after having used *VISOR* (ie, directly after the DA and after 1, 6, and 12 months). Unfortunately, we had to extend our recruitment period because of the COVID-19 pandemic, and consequently, the recruitment period lasted for approximately 12 months (ie, 6 months longer than initially planned). Consequently, given the maximum project duration funded by the Dutch Cancer Society, we did not perform the 12-month follow-up measurements. Therefore, the original primary outcome (ie, 7-day point prevalence after 12 months) had to be adjusted, and the 7-day point prevalence after 6 months was ultimately used as the primary outcome. This change was communicated to and approved by the Dutch Cancer Society. Other changes were not communicated and approved by them as this was not deemed necessary.

Furthermore, because of a technical mistake, some participants who completed *VISOR* but did not complete t=1 (directly after the DA) did not receive an automatic invite for the other follow-ups. When this mistake was discovered, some participants were already lost to follow-up. We dealt with this in two different ways: (1) people who had already missed t=2 but completed the DA in the 3 months before the discovery of the mistake received the invite to participate in t=2 regardless (38/599, 6.3% received this invitation and 3/599, 0.5% made use of this), and (2) people who had already missed t=3 were still invited to participate in t=3 but not t=2 (130/599, 21.7% received the invitation and 5/599, 0.8% made use of this).

Finally, we originally planned to adjust our analyses for covariates that were selected a priori if they were associated with outcomes. However, based on the advice of the involved statistician (SJ), we decided to adjust our analyses for all selected covariates a priori (as described in the study protocol [28]) to keep the covariates consistent across the different models and make the models comparable.

## Results

### Sample

**Figure 3:**
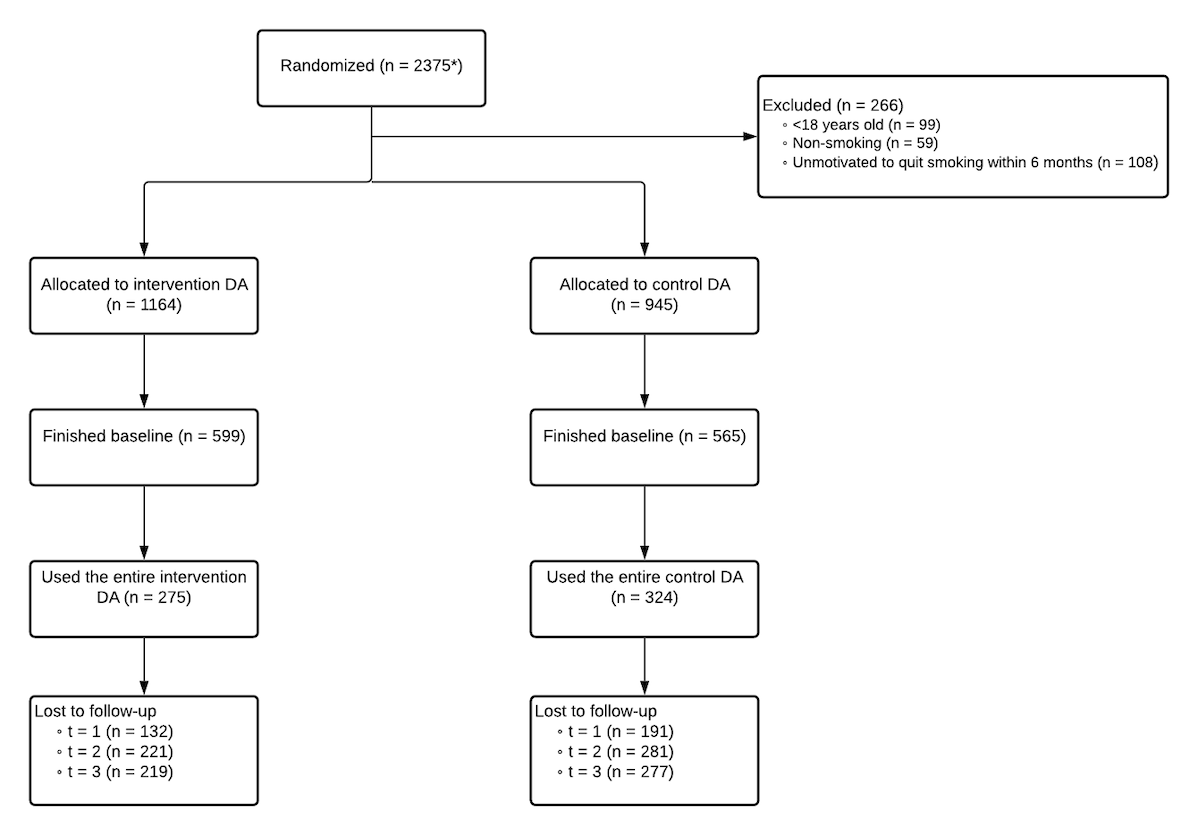
Trial flow adapted from the CONSORT (Consolidated Standards of Reporting Trials) Flow Diagram [57]—the number of participants that were used for the data analyses can be found in the subsequent tables. t=1: immediately after the use of the DA; t=2: 1 month after the use of the DA; t=3: 6 months after the use of the DA; *30 accounts showed duplicate email addresses; of those, only 8 were linked to accounts in both trial arms (ie, multiple accounts using the same email address in each of the trial arms) and finished baseline with >1 account; of those only 2 participants filled in the follow-ups in such a way that they could have a distorting effect on the results (ie, they were first randomized to the intervention group, then to the control group, and then filled in the follow-ups as participants belonging to the control group, although they received the additional intervention elements); therefore, those were adjusted (ie, the randomization variable was changed to 1=intervention). DA: decision aid; t=1: time point 1; t=2: time point 2; t=3: time point 3.

The total sample comprised 2375 participants who were randomized, of whom 1164 (49.01%) completed the baseline questionnaire. Subsequently, of the 2375 participants, 599 (25.22%) completed one of the DAs, 276 (11.62%) filled in t=1 completely, 97 (4.08%) filled in t=2 completely, and 103 (4.34%) filled in t=3 completely. The entire trial flow is shown in Figure 3 [57]*.* The characteristics of the participants who completed the baseline questionnaire are shown in Table 1.

**Table 1:** Characteristics of participants who finished the baseline questionnaire (N=1164).

Participant characteristics			Entire sample	Intervention group (n=599)	Control group (n=565)
**Gender, n (%)**					
	Women		738 (63.4)	372 (62.1)	366 (64.8)
	Men		424 (36.4)	225 (37.6)	199 (35.2)
	Nonbinary		1 (0.09)	1 (0.2)	0 (0.0)
	Prefers not to say		1 (0.09)	1 (0.2)	0 (0.0)
**Age (years), n (%)**					
	18-23		326 (28.01)	173 (28.9)	153 (27.1)
	24-29		155 (13.32)	73 (12.2)	82 (14.5)
	30-100		683 (58.68)	353 (58.9)	330 (58.4)
**Education, n (%)**					
	Low		151 (12.97)	88 (14.7)	63 (11.2)
	Medium		661 (56.78)	321 (53.6)	340 (60.2)
	High		352 (30.24)	190 (31.7)	162 (28.7)
**Tobacco products^a^, n (%)**					
	Cigarettes		1144 (98.28)	586 (97.8)	558 (98.8)
	E-cigarettes^b^		56 (4.81)	28 (4.7)	28 (5)
	Pipe		6 (0.52)	4 (0.7)	2 (0.4)
	Cannabis		42 (3.61)	23 (3.8)	19 (3.4)
	Cigar		17 (1.46)	10 (1.7)	7 (1.2)
	Other		16 (1.37)	8 (1.3)	8 (1.4)
**Tobacco consumption**					
	Total without e-cigarettes (daily), mean (SD)		16.12 (8.64)	16.22 (9.09)	16.00 (8.13)
	**e-Cigarettes only^c^, n (%)**				
		Less than monthly	6 (10.7)	6 (21.4)	0 (0.0)
		Less than weekly but at least once per month	10 (17.9)	2 (7.1)	8 (28.6)
		Less than daily but at least once per week	12 (21.4)	7 (25.0)	5 (17.9)
		Daily but not multiple times	4 (7.1)	3 (10.7)	1 (3.6)
		Multiple times per day	24 (42.9)	10 (35.7)	14 (50.0)
**Smoking cessation behavior**					
	Ever smoking cessation attempt, n (%)		1032 (88.66)	522 (87.1)	510 (90.3)
	Smoking cessation attempts (lasting 24 hours), mean (SD)^d^		4.19 (8.76)	3.99 (9.31)	4.40 (8.16)
	**Cessation assistance use in the past 6 months^e^, n (%)**				
		Evidence based	169 (14.52)	82 (13.7)	87 (15.4)
		Nonevidence based	23 (1.98)	6 (1.0)	17 (3.0)
**Stage of decision-making**					
	Has not begun to think about the choices, n (%)		185 (15.89)	91 (15.2)	94 (16.6)
	Has not begun to think about the choices but is interested in doing so, n (%)		288 (24.74)	147 (24.5)	141 (25.0)
	Is considering the options now, n (%)		404 (34.71)	211 (35.2)	193 (34.2)
	Is close to selecting an option, n (%)		87 (7.47)	45 (7.5)	42 (7.4)
	Already made a decision but is still willing to reconsider, n (%)		124 (10.65)	69 (11.5)	55 (9.7)
	Has already made a decision and is unlikely to change their mind, n (%)		76 (6.53)	36 (6.0)	40 (7.1)
	Values, mean (SD)		2.92 (1.4)	2.94 (1.39)	2.90 (1.42)
	FTND-R^f^, mean (SD)		6.66 (3.30)	6.59 (3.35)	6.75 (3.25)

^a^Selecting multiple products was possible.

^b^All dual users.

^c^Percentages refer to e-cigarette users only.

^d^Excluding extreme outliers ≥1000 and participants who had never attempted to stop smoking before.

^e^At least one, can be multiple; percentages >100% are because of rounding.

^f^FTND-R: Revised Fagerström Test for Nicotine Dependence.

### Attrition

#### Nonuse Attrition

Comparisons of the participants who did not complete one of the DAs and those who did showed significant differences in group allocation (*χ*^2^_1_ [N=1164]=15.2; P<.001), age (*χ*^2^_2_ [N=1164]=78.3; P<.001), level of education *χ*^2^_2_ [N=1164]=12.5; P=.002), whether people used other tobacco products (*χ*^2^_1_ [N=1164]=4.6; P=.03), the average number of cessation attempts (*W*=121,312; P=.02), and stage of decision-making at baseline (*W*=153,400; P=.004). All comparisons can be found in Multimedia Appendix 5. In the logistic regression model, having been allocated to the intervention group (odds ratio [OR] 1.65, 95% CI 1.28-2.14; P<.001), being aged between 24 and 29 years (compared with 18-23 years; OR 0.60, 95% CI 0.38-0.93; P=.02), being aged between 30 and 100 years (compared with 18-23 years; OR 0.29, 95% CI 0.21-0.39; P<.001), and a high (compared with a low) level of education (OR 0.64, 95% CI 0.42-0.99; P=.046) remained significant; that is, participants in the intervention group were more likely *not* to complete *VISOR*, whereas those aged 24 to 100 years (compared with 18-23 years) and those with a high (compared with a low) level of education were more likely to complete *VISOR*.

#### Dropout Attrition

Comparisons of the participants who did not complete t=1 and those who did showed significant differences in group allocation (*χ*^2^_1_ [N=599]=7.2; P=.01), gender (*χ*^2^_1_ [N=598]=5.9; P=.02), and stage of decision-making (*W*=37,942; P=.001). All comparisons can be found in Multimedia Appendix 6. All variables remained significant in the logistic regression model: having been allocated to the intervention group (OR 0.65, 95% CI 0.47-0.90; P=.01), men compared with women (OR 0.65, 95% CI 0.46-0.92; P=.01), and stage of decision-making (OR 0.82, 95% CI 0.72-0.92; P=.001); that is, participants in the intervention group, men (compared with women) and those in higher stages of decision-making were less likely to drop out.

Comparisons of the participants who did not complete t=2 and those who did showed significant differences in group allocation (*χ*^2^_1_ [N=599]=4.4; P=.04) and stage of decision-making (*W*=21,158; P=.03). All comparisons can be found in Multimedia Appendix 7. In the logistic regression model, having been allocated to the intervention group remained significant (OR 0.63, 95% CI 0.41-0.98; P=.04); that is, participants in the intervention group were less likely to drop out.

Comparisons of the participants who did not complete t=3 and those who did showed significant differences only in the stage of decision-making (*W*=21,601; P=.01), which also remained significant in the logistic regression (OR 0.84, 95% CI 0.72-0.97; P=.02); that is, participants in higher stages of decision-making were less likely to drop out. All comparisons can be found in Multimedia Appendix 8.

As next to the demographic variables (which were already planned as covariates for all analyses) and group allocation, only the stage of decision-making was most consistently associated with dropout; we decided to include the stage of decision-making as a covariate for all analyses as well—and not only decisional conflict as planned in the protocol [28].

### Hypotheses Testing

#### Hypothesis 1a: Smoking Cessation After 1 Month

Although it was observed that more participants stopped smoking in the intervention group (15/55, 27% of the respondents) than in the control group (7/46, 15% of the respondents) after 1 month, the intervention did not result in a significant effect on smoking cessation in the complete case analyses (OR 2.09, 95% CI 0.79 to infinity [+], crude P=.07; OR 1.93, 95% CI 0.64 to infinity [+], adjusted P=.13) or the MI analyses (OR 1.26, 95% CI 0.63 to infinity [+], crude P=.25; OR 1.35, 95% CI 0.57 to infinity [+], adjusted P=.24). However, in the worst-case scenario, effects in favor of the intervention group were observed (OR 2.61, 95% CI 1.08 to infinity [+], crude P=.02; OR 2.71, 95% CI 1.09 to infinity [+], adjusted P=.02). In 2 of the 3 adjusted models (ie, complete cases and worst-case scenario), the only (other) variable that had a significant effect on smoking cessation was the stage of decision-making (complete case analysis: OR 1.83, 95% CI 1.24-2.85; P=.004; worst-case scenario: OR 1.63, 95% CI 1.22-2.22; P=.001). In the MI scenario, none of the included variables had a significant effect on smoking cessation rates after 1 month. Therefore, hypothesis 1a could only be confirmed in the worst-case scenario. Table 2 presents the results and more information.

**Table 2:** Results of logistic regression for hypothesis 1a and 1b: smoking cessation after 1 month and 6 months.

Time point and variable				B (SE)		P value		Odds ratio (95% CI)
**After 1 month**								
	**Complete cases (crude)^a^**							
		Intercept	−1.72 (0.41)		<.001		N/A^b^	
		Group allocation (intervention)	0.74 (0.51)		.07^c^		2.09 (0.79 to infinity)	
	**Complete cases (adjusted)^d^**							
		Intercept	−3.30 (1.67)		.048		N/A	
		Group allocation (intervention)	0.66 (0.58)		.13^c^		1.93 (0.64 to infinity)	
		Age (24-29 years)	−1.53 (1.30)		.24		0.22 (0.01 to 2.13)	
		Age (30-100 years)	−1.27 (0.70)		.07		0.28 (0.07 to 1.12)	
		Gender (men)	0.21 (0.57)		.72		1.23 (0.39 to 3.76)	
		Education (medium)	0.01 (1.12)		.99		1.01 (0.12 to 10.67)	
		Education (high)	0.36 (1.11)		.74		1.44 (0.17 to 14.87)	
		FTND-R^e^	0.04 (0.08)		.65		1.04 (0.88 to 1.22)	
		Stage of decision-making	0.61 (0.21)		.004		1.83 (1.24 to 2.85)	
	**Worst-case scenario (crude)^f^**							
		Intercept	−3.81 (0.38)		<.001		N/A	
		Group allocation (intervention)	0.96 (0.47)		.02^c^		2.61 (1.08 to infinity)	
	**Worst-case scenario (adjusted)^g^**							
		Intercept	−5.21 (1.23)		<.001		N/A	
		Group allocation (intervention)	1.0 (0.48)		.02^c^		2.71 (1.09 to infinity)	
		Age (24-29 years)	−1.75 (1.11)		.11		0.17 (0.01 to 1.08)	
		Age (30-100 years)	−0.94 (0.53)		.08		0.39 (0.14 to 1.17)	
		Gender (men)	0.03 (0.47)		.94		1.04 (0.40 to 2.53)	
		Education (medium)	0.32 (0.83)		.69		1.38 (0.32 to 9.64)	
		Education (high)	0.89 (0.84)		.29		2.44 (0.55 to 17.32)	
		FTND-R	−0.01 (0.07)		.86		0.99 (0.86 to 1.13)	
		Stage of decision-making	0.49 (0.15)		.001		1.63 (1.22 to 2.22)	
	**Multiple imputations (crude)^h^**							
		Intercept	−0.82 (0.43)		.07		N/A	
		Group allocation (intervention)	0.23 (0.35)		.25^c^		1.26 (0.63 to infinity)	
	**Multiple imputations (adjusted)^i^**							
		Intercept	−1.66 (1.41)		.24		N/A	
		Group allocation (intervention)	0.30 (0.43)		.24^c^		1.35 (0.57 to infinity)	
		Age (24-29 years)	−0.95 (1.00)		.35		0.39 (0.05 to 2.88)	
		Age (30-100 years)	−0.75 (0.59)		.21		0.47 (0.15 to 1.54)	
		Gender (men)	−0.06 (0.51)		.90		0.94 (0.34 to 2.62)	
		Education (medium)	0.01 (0.99)		.99		1.01 (0.14 to 7.40)	
		Education (high)	0.33 (0.96)		.74		1.39 (0.20 to 9.64)	
		FTND-R	0.02 (0.06)		.72		1.02 (0.90 to 1.16)	
		Stage of decision-making	0.35 (0.21)		.10		1.42 (0.93 to 2.17)	
**After 6 months**								
	**Complete cases (crude)^j^**							
		Intercept	−1.0 (0.31)		<.01		N/A	
		Group allocation (intervention)	0.45 (0.41)		.14^c^		1.56 (0.71 to infinity)	
	**Complete cases (adjusted)^k^**							
		Intercept	−0.50 (1.09)		.65		N/A	
		Group allocation (intervention)	0.24 (0.45)		.30^c^		1.27 (0.52 to infinity)	
		Age (24-29 years)	−1.82 (1.19)		.13		0.16 (0.01 to 1.24)	
		Age (30-100 years)	−0.49 (0.58)		.40		0.61 (0.19 to 1.94)	
		Gender (men)	0.74 (0.45)		.10		2.09 (0.87 to 5.06)	
		Education (medium)	−0.80 (0.75)		.29		0.45 (0.10 to 2.04)	
		Education (high)	−0.46 (0.78)		.55		0.63 (0.13 to 2.98)	
		FTND-R	−0.10 (0.07)		.12		0.90 (0.78 to 1.03)	
		Stage of decision-making	0.31 (0.16)		.05		1.36 (1.00 to 1.88)	
	**Worst-case scenario (crude)^l^**							
		Intercept	−3.10 (0.27)		<.001		N/A	
		Group allocation (intervention)	0.70 (0.35)		.02^c^		2.02 (1.03 to infinity)	
	**Worst-case scenario (adjusted)^m^**							
		Intercept	−3.04 (0.86)		<.001		N/A	
		Group allocation (intervention)	0.62 (0.36)		.04^c^		1.85 (0.92 to infinity)	
		Age (24-29 years)	−1.80 (1.08)		.10		0.17 (0.01 to 0.96)	
		Age (30-100 years)	−0.17 (0.44)		.69		0.84 (0.36 to 2.09)	
		Gender (men)	0.64 (0.35)		.07		1.90 (0.95 to 3.79)	
		Education (medium)	−0.36 (0.56)		.52		0.70 (0.25 to 2.32)	
		Education (high)	−0.13 (0.58)		.82		0.88 (0.29 to 3.0)	
		FTND-R	−0.14 (0.06)		.02		0.87 (0.78 to 0.98)	
		Stage of decision-making	0.32 (0.12)		.01		1.37 (1.09 to 1.72)	
	**Multiple imputations (crude)^n^**							
		Intercept	−0.78 (0.31)		.01		N/A	
		Group allocation (intervention)	0.30 (0.41)		.23^c^		1.35 (0.59 to infinity)	
	**Multiple imputations (adjusted)^o^**							
		Intercept	0.14 (1.06)		.89		N/A	
		Group allocation (intervention)	0.19 (0.43)		.33^c^		1.21 (0.52 to infinity)	
		Age (24-29 years)	−1.66 (1.07)		.13		0.19 (0.02 to 1.62)	
		Age (30-100 years)	−0.45 (0.45)		.32		0.64 (0.26 to 1.56)	
		Gender (men)	0.65 (0.50)		.20		1.91 (0.70 to 5.21)	
		Education (medium)	−0.98 (0.72)		.18		0.37 (0.09 to 1.57)	
		Education (high)	−0.56 (0.75)		.46		0.57 (0.13 to 2.57)	
		FTND-R	−0.10 (0.06)		.09		0.91 (0.81 to 1.02)	
		Stage of decision-making	0.22 (0.16)		.18		1.24 (0.90 to 1.71)	

^a^*R*^2^=0.02 (Hosmer-Lemeshow), 0.02 (Cox-Snell), 0.03 (Nagelkerke); *χ*^2^_1_=2.2; P=.14; 101/1164, 8.68%.

^b^N/A: not applicable.

^c^1-sided.

^d^*R*^2^=0.14 (Hosmer-Lemeshow), 0.13 (Cox-Snell), 0.21 (Nagelkerke); *χ*^2^_7_=12.3; P=.09 (compared with the crude model), 101/1164, 8.68%

^e^FTND-R: Revised Fagerström Test for Nicotine Dependence.

^f^*R*^2^=0.02 (Hosmer-Lemeshow), 0.01 (Cox-Snell), 0.03 (Nagelkerke); *χ*^2^_1_=4.6; P=.03; 599/1164, 51.46%.

^g^*R*^2^=0.11 (Hosmer-Lemeshow), 0.03 (Cox-Snell), 0.13 (Nagelkerke); *χ*^2^_7_=16.2; P=.02 (compared with the crude model, excluding the nonbinary participant), 598/1164, 51.37%.

^h^*R*^2^=0.01 (Hosmer-Lemeshow), 0.01 (Cox-Snell), 0.01 (Nagelkerke); *χ*^2^_1_=4.4; P=.52; 599/1164, 51.46%.

^i^*R*^2^=0.1 (Hosmer-Lemeshow), 0.11 (Cox-Snell), 0.16 (Nagelkerke); *χ*^2^_7_=70.1; P=.58 (compared with the crude model, excluding the nonbinary participant), 598/1164, 51.37%.

^j^*R*^2^=0.01 (Hosmer-Lemeshow), 0.01 (Cox-Snell), 0.01 (Nagelkerke); *χ*^2^_1_=1.21; P=.27; 115/1164, 9.88%.

^k^*R*^2^=0.11 (Hosmer-Lemeshow), 0.12 (Cox-Snell), 0.17 (Nagelkerke); *χ*^2^_7_=14.1; P=.05 (compared with the crude model), 115/1164, 9.88%.

^l^*R*^2^=0.02 (Hosmer-Lemeshow), 0.01 (Cox-Snell), 0.02 (Nagelkerke); *χ*^2^_1_=4.2; P=.04; 599/1164, 51.46%.

^m^*R*^2^=0.09 (Hosmer-Lemeshow), 0.04 (Cox-Snell), 0.11 (Nagelkerke); *χ*^2^_7_=20.9; P<.01 (compared with the crude model, excluding the nonbinary participant), 598/1164, 51.37%.

^n^*R*^2^=0.01 (Hosmer-Lemeshow), 0.01 (Cox-Snell), 0.02 (Nagelkerke); *χ*^2^_1_=7.2; P=.48; 599/1164, 51.46%.

^o^*R*^2^=0.12 (Hosmer-Lemeshow), 0.14 (Cox-Snell), 0.19 (Nagelkerke); *χ*^2^_7_=77.3;P=.24 (compared with the crude model, excluding the nonbinary participant), 598/1164, 51.37%.

#### Hypothesis 1b: Smoking Cessation After 6 Months

Although it was again observed that after 6 months, more participants reported having stopped smoking in the intervention group (23/63, 37% of the respondents) than in the control group (14/52, 27% of the respondents), the intervention did not result in a significant effect on smoking cessation in the complete case analyses (OR 1.56, 95% CI 0.71 to infinity [+], crude P=.14; OR 1.27, 95% CI 0.52 to infinity [+], adjusted P=.30) or the MI analyses (OR 1.35, 95% CI 0.59 to infinity [+], crude P=.23; OR 1.21, 95% CI 0.52 to infinity [+], adjusted P=.33). Similar to the results based on data collected after 1 month, effects in favor of the intervention group were observed in the worst-case scenario (OR 2.02, 95% CI 1.03 to infinity [+], crude P=.02; OR 1.85, 95% CI 0.92 to infinity [+], adjusted P=.04). In the worst-case scenario, the FTND-R (OR 0.87, 95% CI 0.78-0.98; P=.02; ie, nicotine dependence) and stage of decision-making (OR 1.37, 95% CI 1.09-1.72; P=.01) had a significant effect on smoking cessation rates. However, the FTND-R violated the linearity assumption in this model, but excluding it did not change the conclusions in relation to the primary outcome (Multimedia Appendix 9). In the complete case analyses and the MI scenario, none of the included variables had a significant effect on smoking cessation rates after 6 months. Therefore, hypothesis 1b could only be confirmed in the worst-case scenario. Table 2 presents the results and more information.

#### Hypothesis 2a: Use of Evidence-Based Cessation Assistance After 1 Month

Although, after 1 month, more people in the intervention group reported to have used an evidence-based cessation assistance tool (26/56, 46% of the respondents) than those in the control group (18/47, 38% of the respondents), the intervention did not result in a significant effect on the uptake of evidence-based cessation assistance in the complete case analysis (OR 1.40, 95% CI 0.64 to infinity [+], crude P=.20; OR 1.42, 95% CI 0.60 to infinity [+], adjusted P=.21) or the MI analyses (OR 1.25, 95% CI 0.69 to infinity [+], crude P=.23; OR 1.42, 95% CI 0.61 to infinity [+], adjusted P=.20). Similar to smoking cessation, effects in favor of the intervention group appeared in the worst-case scenario but only in the crude model (OR 1.78, 95% CI 0.96 to infinity [+], crude P=.04; OR 1.68, 95% CI 0.89 to infinity [+], adjusted P=.05). In the adjusted models, only the stage of decision-making had a significant effect on the outcome (in the complete case analysis, OR 1.53, 95% CI 1.11-2.19, P=.01; in the worst-case scenario, OR 1.36, 95% CI 1.10-1.68; P=.005; in the MI analysis, OR 1.47, 95% CI 1.07-2.02; P=.02). Therefore, hypothesis 2a could only be partially confirmed in the worst-case scenario. Table 3 presents the results and more information.

**Table 3:** Results of logistic regression for hypothesis 2a and 2b: use of evidence-based cessation assistance after 1 month and 6 months.

Time point and variable				B (SE)		P value		Odds ratio (95% CI)
**After 1 month**								
	**Complete cases (crude)^a^**							
		Intercept	−0.48 (0.30)		.11		N/A^b^	
		Group allocation (intervention)	0.33 (0.40)		.20^c^		1.40 (0.64 to infinity)	
	**Complete cases (adjusted)^d^**							
		Intercept	−2.76 (0.85)		<.01		N/A	
		Group allocation (intervention)	0.35 (0.44)		.21^c^		1.42 (0.60 to infinity)	
		Age (24-29 years)	0.49 (0.96)		.61		1.62 (0.23 to 10.65)	
		Age (30-100 years)	0.51 (0.61)		.40		1.66 (0.52 to 5.74)	
		Gender (men)	0.10 (0.46)		.82		1.11 (0.45 to 2.76)	
		Education (high)	−0.29 (0.45)		.52		0.75 (0.30 to 1.80)	
		FTND-R^e^	0.08 (0.06)		.19		1.09 (0.96 to 1.24)	
		Stage of decision-making	0.43 (0.17)		.01		1.53 (1.11 to 2.19)	
	**Worst-case scenario (crude)^f^**							
		Intercept	−2.83 (0.24)		<.001		N/A	
		Group allocation (intervention)	0.57 (0.32)		.04^c^		1.78 (0.96 to infinity)	
	**Worst-case scenario (adjusted)^g^**							
		Intercept	−3.98 (0.82)		<.001		N/A	
		Group allocation (intervention)	0.52 (0.32)		.05^c^		1.68 (0.89 to infinity)	
		Age (24-29 years)	−0.34 (0.74)		.65		0.71 (0.14 to 2.91)	
		Age (30-100 years)	0.24 (0.48)		.62		1.27 (0.53 to 3.56)	
		Gender (men)	0.05 (0.33)		.89		1.05 (0.54 to 1.98)	
		Education (medium)	−0.33 (0.48)		.49		0.72 (0.29 to 1.97)	
		Education (high)	−0.06 (0.50)		.90		0.94 (0.36 to 2.66)	
		FTND-R	0.03 (0.05)		.57		1.03 (0.93 to 1.14)	
		Stage of decision-making	0.30 (0.11)		.005		1.36 (1.10 to 1.68)	
	**Multiple imputations (crude)^h^**							
		Intercept	−0.55 (0.34)		.11		N/A	
		Group allocation (intervention)	0.22 (0.30)		.23^c^		1.25 (0.69 to infinity)	
	**Multiple imputations (adjusted)^i^**							
		Intercept	−2.71 (0.86)		<.01		N/A	
		Group allocation (intervention)	0.35 (0.42)		.20^c^		1.42 (0.61 to infinity)	
		Age (24-29 years)	0.33 (0.86)		.70		1.39 (0.25 to 7.72)	
		Age (30-100 years)	0.45 (0.52)		.39		1.57 (0.56 to 4.43)	
		Gender (men)	0.12 (0.46)		.79		1.13 (0.45 to 2.85)	
		Education (high)	−0.20 (0.44)		.65		0.82 (0.34 to 1.97)	
		FTND-R	0.09 (0.06)		.11		1.10 (0.98 to 1.23)	
		Stage of decision-making	0.39 (0.16)		.02		1.47 (1.07 to 2.02)	
**After 6 months**								
	**Complete cases (crude)^j^**							
		Intercept	0.08 (0.28)		.78		N/A	
		Group allocation (intervention)	0.42 (0.39)		.14^c^		1.53 (0.72 to infinity)	
	**Complete cases (adjusted)^k^**							
		Intercept	−1.14 (1.13)		.31		N/A	
		Group allocation (intervention)	0.54 (0.43)		.11^c^		1.71 (0.74 to infinity)	
		Age (24-29 years)	−1.06 (0.87)		.22		0.35 (0.06 to 1.82)	
		Age (30-100 years)	0.06 (0.56)		.92		1.06 (0.35 to 3.16)	
		Gender (men)	−0.15 (0.43)		.73		0.86 (0.37 to 2.01)	
		Education (medium)	−0.01 (0.78)		.99		0.99 (0.19 to 4.36)	
		Education (high)	−0.06 (0.81)		.94		0.94 (0.17 to 4.46)	
		FTND-R	0.10 (0.06)		.12		1.10 (0.98 to 1.25)	
		Stage of decision-making	0.21 (0.15)		.17		1.24 (0.92 to 1.68)	
	**Worst-case scenario (crude)^l^**							
		Intercept	−2.44 (0.20)		<.001		N/A	
		Group allocation (intervention)	0.61 (0.27)		.01^c^		1.84 (1.09 to infinity)	
	**Worst-case scenario (adjusted)^m^**							
		Intercept	−3.48 (0.70)		<.001		N/A	
		Group allocation (intervention)	0.59 (0.27)		.02^c^		1.80 (1.06 to infinity)	
		Age (24-29 years)	−0.94 (0.68)		.17		0.39 (0.08 to 1.34)	
		Age (30-100 years)	0.10 (0.38)		.80		1.10 (0.54 to 2.40)	
		Gender (men)	0.02 (0.28)		.94		1.02 (0.58 to 1.76)	
		Education (medium)	0.27 (0.45)		.55		1.31 (0.57 to 3.44)	
		Education (high)	0.22 (0.48)		.65		1.25 (0.50 to 3.43)	
		FTND-R	0.01 (0.04)		.87		1.01 (0.92 to 1.10)	
		Stage of decision-making	0.24 (0.09)		.01		1.27 (1.06 to 1.53)	
	**Multiple imputations (crude)^n^**							
		Intercept	−0.09 (0.25)		.73		N/A	
		Group allocation (intervention)	0.54 (0.37)		.07^c^		1.72 (0.83 to infinity)	
	**Multiple imputations (adjusted)^o^**							
		Intercept	−1.11 (1.08)		.31		N/A	
		Group allocation (intervention)	0.50 (0.43)		.13^c^		1.65 (0.69 to infinity)	
		Age (24-29 years)	−0.84 (0.78)		.29		0.43 (0.09 to 2.06)	
		Age (30-100 years)	0.15 (0.47)		.76		1.16 (0.45 to 2.96)	
		Gender (men)	−0.20 (0.47)		.67		0.82 (0.32 to 2.10)	
		Education (medium)	−0.14 (0.63)		.83		0.87 (0.25 to 3.04)	
		Education (high)	−0.24 (0.68)		.73		0.79 (0.20 to 3.05)	
		FTND-R	0.08 (0.06)		.19		1.09 (0.96 to 1.23)	
		Stage of decision-making	0.24 (0.15)		.12		1.27 (0.94 to 1.72)	

^a^*R*^2^=0.005 (Hosmer-Lemeshow), 0.01 (Cox-Snell), 0.01 (Nagelkerke); *χ*^2^_1_=0.7; P=.41; 103/1164, 8.85%.

^b^N/A: not applicable.

^c^1-sided.

^d^*R*^2^=0.09 (Hosmer-Lemeshow), 0.11 (Cox-Snell), 0.15 (Nagelkerke); *χ*^2^_6_=11.7; P=.07 (compared with the crude model), 103/1164, 8.85%.

^e^FTND-R: Revised Fagerström Test for Nicotine Dependence.

^f^*R*^2^=0.01 (Hosmer-Lemeshow), 0.01 (Cox-Snell), 0.01 (Nagelkerke); *χ*^2^_1_=3.3; P=.07; 599/1164, 51.46%.

^g^*R*^2^=0.05 (Hosmer-Lemeshow), 0.02 (Cox-Snell), 0.06 (Nagelkerke); *χ*^2^_7_=11.2; P=.13 (compared with the crude model, excluding the nonbinary participant), 598/1164, 51.37%.

^h^*R*^2^=0.005 (Hosmer-Lemeshow), 0.01 (Cox-Snell), 0.01 (Nagelkerke); *χ*^2^_1_=3.8; P=.46; 599/1164, 51.46%.

^i^*R*^2^=0.11 (Hosmer-Lemeshow), 0.13 (Cox-Snell), 0.18 (Nagelkerke); *χ*^2^_6_=77.2; P=.13 (compared with the crude model, excluding the nonbinary participant), 598/1164, 51.37%.

^j^*R*^2^=0.01 (Hosmer-Lemeshow), 0.01 (Cox-Snell), 0.01 (Nagelkerke); *χ*^2^_1_=1.2; P=.27; 111/1164, 9.54%.

^k^*R*^2^=0.07 (Hosmer-Lemeshow), 0.09 (Cox-Snell), 0.12 (Nagelkerke); *χ*^2^_7_=8.8; P=.26 (compared with the crude model), 111/1164, 9.54%.

^l^*R*^2^=0.01 (Hosmer-Lemeshow), 0.01 (Cox-Snell), 0.02 (Nagelkerke); *χ*^2^_1_=5.2; P=.02; 599/1164, 51.46%.

^m^*R*^2^=0.04 (Hosmer-Lemeshow), 0.03 (Cox-Snell), 0.05 (Nagelkerke); *χ*^2^_7_=10.8; P=.15 (compared with the crude model, excluding the nonbinary participant), 598/1164, 51.37%.

^n^*R*^2^=0.02 (Hosmer-Lemeshow), 0.02 (Cox-Snell), 0.03 (Nagelkerke); *χ*^2^_1_=14.1; P=.14; 599/1164, 51.46%.

^o^*R*^2^=0.10 (Hosmer-Lemeshow), 0.12 (Cox-Snell), 0.16 (Nagelkerke); *χ*^2^_7_=65.3; P=.37 (compared with the crude model, excluding the nonbinary participant), 598/1164, 51.37%.

#### Hypothesis 2b: Use of Evidence-Based Cessation Assistance After 6 Months

Similarly, although, after 6 months, more people in the intervention group reported using an evidence-based cessation assistance tool (38/61, 62% of the respondents) than those in the control group (26/50, 52% of the respondents), the intervention did not result in a significant effect on the uptake of evidence-based cessation assistance in the complete case analysis (OR 1.53, 95% CI 0.72 to infinity [+], crude P=.14; OR 1.71, 95% CI 0.74 to infinity [+], adjusted P=.11) or the MI analysis (OR 1.72, 95% CI 0.83 to infinity [+], crude P=.07; OR 1.65, 95% CI 0.69 to infinity [+], adjusted P=.13). However, in the worst-case scenario, effects in favor of the intervention group emerged (OR 1.84, 95% CI 1.09 to infinity [+], crude P=.01; OR 1.80, 95% CI 1.06 to infinity [+], adjusted P=.02). In the adjusted models, the stage of decision-making had a significant effect on the outcome but only in the worst-case scenario (OR 1.27, 95% CI 1.06-1.53; P=.01). Therefore, hypothesis 2b could only be confirmed in the worst-case scenario. Table 3 presents the results and more information.

#### Hypothesis 3: Decisional Conflict Immediately After the DA

Despite the small difference in averages between the intervention (mean 39.29, SD 25.00) and control groups (mean 41.17, SD 25.23), the intervention had no significant effect on the decisional conflict in either the complete case analysis (β=−0.04, crude P=.25; β=−0.05, adjusted P=.20) or MI analysis (β=−0.05, crude P=.18; β=−0.05, adjusted P=.22). In the adjusted models, only the stage of decision-making (β=−0.15, P=.005 in the complete case analysis; β=−0.14, P=.01 in the MI analysis), being aged between 30 and 100 years (compared with 18-23 years) (only in the MI analysis, β=−0.06, P=.045), and a high level of education (β=−0.27, P=.002 in the complete case analysis; β=−0.25, P=.005 in the MI analysis) had a significant effect on the outcome. All of them had a negative effect; that is, people who reported a higher stage of decision-making, participants aged between 30 and 100 years old (compared with 18-23 years), and participants with a high level of education (compared with participants with a low level of education) experienced less decisional conflict. Therefore, hypothesis 3 could not be confirmed. Table 4 presents the results and more information.

**Table 4:** Results of linear regression for hypothesis 3: decisional conflict immediately after using the decision aid^a^.

Case analysis			B (95% CI)		SE		β		P value
**Complete cases (crude)^b^**									
	Intercept	41.17 (37.43 to 44.92)		1.90		N/A^c^		<.001	
	Group allocation (intervention)	−1.89 (infinity to 3.52)		2.75		−0.04		.25^d^	
**Complete cases (adjusted)^e^**									
	Intercept	62.69 (50.44 to 74.94)		6.23		N/A		<.001	
	Group allocation (intervention)	−2.31 (infinity to 3.03)		2.71		−0.05		.20^d^	
	Age (24-29 years)	−4.05 (−15.08 to 6.98)		5.61		−0.05		.47	
	Age (30-100 years)	−6.60 (−14.07 to 0.86)		3.80		−0.11		.08	
	Gender (men)	2.51 (−2.97 to 7.99)		2.79		.05		.37	
	Education (medium)	−6.44 (−15.12 to 2.24)		4.41		−0.13		.15	
	Education (high)	−13.91 (−22.85 to −4.98)		4.54		−0.27		.002	
	Stage of decision-making	−2.69 (−4.55 to −0.84)		0.94		−0.15		.005	
**Multiple imputations (crude)^f^**									
	Intercept	42.36 (38.49 to 46.23)		1.96		N/A		<.001	
	Group allocation (intervention)	−2.53 (infinity to 2.83)		2.71		−0.05		.18^d^	
**Multiple imputations (adjusted)^g^**									
	Intercept	62.88 (50.50 to 75.25)		6.25		N/A		<.001	
	Group allocation (intervention)	−2.32 (infinity to 3.67)		3.02		−0.05		.22^d^	
	Age (24-29 years)	−4.73 (−14.93 to 5.48)		5.16		−0.06		.36	
	Age (30-100 years)	−7.47 (−14.78 to −0.16)		3.69		−0.14		.045	
	Gender (men)	2.27 (−3.26 to 7.79)		2.80		.04		.42	
	Education (medium)	−6.13 (−15.34 to 3.08)		4.65		−0.12		.19	
	Education (high)	−12.99 (−21.90 to −4.08)		4.51		−0.25		.005	
	Stage of decision-making	−2.52 (−4.51 to −0.52)		1.01		−0.14		.01	

^a^It should be noted that the residuals were not perfectly normally distributed in the models; this was especially apparent in the crude model (complete cases). However, overall, the skew was not highly substantial.

^b^Multiple *R*^2^=0.001; P=.49; 335/1164, 28.78%.

^c^N/A: not applicable.

^d^1-sided.

^e^Multiple *R*^2^=0.07; P<.001 (compared with the crude model), 335/1164, 28.78%.

^f^Multiple *R*^2^=0.003; P=.36; 599/1164, 51.46%.

^g^Multiple *R*^2^=0.08; P=.001 (compared with the crude model, excluding the nonbinary participant), 598/1164, 51.37%.

## Discussion

### Principal Findings

The aim of this paper was to report the effects of adding an explicit VCM with computer-tailored advice to a smoking cessation DA (*VISOR*) on both smoking cessation outcomes and decisional conflict. Contrary to our expectations, we did not find any effect on decisional conflict. In addition, although the worst-case scenarios might suggest an effect on smoking cessation rates and cessation assistance uptake, this finding was not replicated in either the complete case analyses or MI analyses. Moreover, given the fact that Blankers et al [58] showed that analyses based on penalized imputation can be biased when missingness is unbalanced between trial arms (as in our case), we cannot confidently speak of the effects on smoking cessation success and cessation assistance uptake, despite the suggestion of effects in the worst-case scenarios. That said, all the significant and nonsignificant effects that were found were in the expected direction; that is, participants in the intervention group showed more smoking cessation, more evidence-based cessation assistance uptake, and less decisional conflict. A summary of the main findings with respect to the primary and secondary outcomes can be found in Textbox 2.

However, based on these findings, it may be too early to declare the addition of explicit VCMs and computer-tailored advice as ineffective to smoking cessation DAs. Previous studies have consistently found effects in favor of explicit VCMs on value-congruent decision-making in other contexts, especially when combined with computer-tailored advice [22,25]. That said, we were the first (to the best of our knowledge) to test this in a smoking cessation context. We would also like to emphasize that our findings do not imply that smoking cessation DAs are ineffective, as we did not compare a smoking cessation DA with usual care, another intervention, or no intervention but rather compared 2 different versions of one and the same DA: one with a VCM and paired computer-tailored advice and one without. In fact, even the control group in this RCT exceeded the smoking cessation rates achieved by the only other Dutch smoking cessation DA described in the literature [17]. However, this study showed that adding explicit VCMs paired with computer-tailored advice might not always be a good idea, as nonuse attrition was 34.19% higher in the intervention group than in the control group. In addition, our nonsignificant findings entail some caveats. In this discussion, we will focus on the two most likely explanations for the lack of effects we found: (1) we were unable to find an effect because of a lack of power, and (2) there truly was no effect. In addition, we also describe the implications of these 2 explanations and how readers might benefit from the insights generated during our project.

**Summary of the main effects.**

**Summary of the main effects**

- Only the scenario in which we assumed that all participants who dropped out continued to smoke suggests that adding an explicit value clarification method and computer-tailored advice to a digital smoking cessation decision aid has statistically significant effects on smoking cessation rates and evidence-based cessation assistance uptake.
- All effects went in the hypothesized directions in all scenarios; that is, in the group that received the additional elements, more participants quit, more participants used evidence-based smoking cessation assistance, and participants experienced less decisional conflict.

### Lack of Statistical Power

The most likely explanation for the lack of significant effects was the lack of statistical power. During this trial, we faced a relatively high attrition rate (as is typical for digital interventions [53]), and ultimately, our trial was widely underpowered as even the MI analyses were underpowered to conduct our analyses. On the basis of our power analyses, we planned to include 1592 smokers at baseline, accounting for 50% of attrition. Ultimately, we randomized 2375 participants but had already lost 1211 (50.99%) individuals from this group between randomization and the end of the baseline questionnaire. Within this group, we subsequently lost 48.54% (565/1164) of participants to nonuse attrition and, among the participants invited to follow-up, 53.92% to 83.8% of participants to drop out attrition. Therefore, attrition was much higher than originally anticipated. Although replication of our study with a larger sample is recommended, researchers and DA developers can learn from the reasons for attrition uncovered in this project (eg, use time), as will be explained in the following sections.

Of the allocated participants, only 23.63% (275/1164) of the participants allocated to the intervention group completed *VISOR* compared with 34.29% (324/945) of the participants allocated to the control group. Interestingly, the available data provided by the intervention host indicated that the differences in use times between the 2 groups were significantly different. The median use time of the intervention group was approximately 9 minutes, whereas that of the control group was approximately 6 minutes (see the OSF for more information [35]). This difference may have driven the differences in nonuse attrition between the intervention and control groups. Our other findings regarding nonuse attrition also give credence to this explanation as younger participants were more likely to not complete *VISOR*. During the needs assessment conducted to develop *VISOR*, younger participants in particular indicated that relatively short time frames were acceptable for using a smoking cessation DA [29]. Therefore, future research should explore whether VCMs and computer-tailored advice can be designed to be delivered in a shorter manner to alleviate this problem. For example, Witteman et al [25] reported a digital VCM (including computer-tailored advice) that comprised dynamic web sliders representing both values and preferences (ie, the included options). These web sliders were then linked, meaning that for participants who indicated that a particular value was very important to them, the slider representing the preference moved equally. Such a VCM could potentially be much shorter to use as end users do not have to answer multiple statements. However, at this point, this technique is relatively difficult to use for decisions involving multiple options, which is why we were unable to use it within *VISOR*. However, on the basis of our findings, one might also conclude that it is important to spend more time studying user experience components, especially use times. A well-known method of studying this is the think-aloud method (which has also been used to test *VISOR*’s usability [28]). In studies using the think-aloud method, participants are asked to use an intervention while verbalizing their thoughts to uncover the cognitive processes and emotional reactions when using the intervention [59,60].

Interestingly, based on our data, it can also be concluded that we did not experience a recruitment problem (ie, 2109 participants were initially randomized and eligible to participate) but rather a problem of retention—only a small number of participants completed *VISOR* and even fewer participants completed the follow-up questionnaires. Although this is often observed in digital health care interventions [61], it is crucial to find ways of increasing the actual use of digital DAs and retention in DA trials to ensure benefits for users and also reach sufficient statistical power during studies. A way of achieving this would be to embed DAs (such as *VISOR*) in a counseling pathway as the involvement of a professional has been shown to positively influence time spent on websites aimed at improving healthy lifestyles [61]. Concurrently, this might also positively influence retention rates in studies (ie, people returning for follow-up measurements) in which digital DAs are evaluated [62,63]. There could be multiple ways of accomplishing this in the context of *VISOR* (or other digital DAs for that matter): (1) *VISOR* could be used together with a health care professional (eg, a practice nurse) or could be sent to participants before or after a health care consultation in which (the result of) *VISOR* is discussed, or (2) a digital form of counseling could be included in *VISOR* (eg, through the form of videocalls [64] or an automated chatbot [65]). The second approach might be especially promising as it would keep the fully digital and automated nature of *VISOR* intact, thereby still ensuring the optimal reach of the DA. Future research should investigate which modalities are especially beneficial in terms of outcomes and retention and which modalities are preferred by end users themselves.

Related to the problem of retention, an interesting chance finding of our study was that it highlighted the importance of the stage of decision-making in this regard, as this influenced both nonuse and dropout attrition. Interestingly, this influence seemed to disappear (partially) once we corrected for other dropout predictors. The most plausible explanation for this is that different groups within our sample (eg, older compared with younger participants) differed in their stage of decision-making (see the OSF for more information [35]), leading to the stage of decision-making becoming insignificant once all predictors were added in the same model. Interestingly, the stage of decision-making is much less routinely assessed than decisional conflict; to illustrate, the validation article of the original Decisional Conflict Scale [9] has been cited >2000 times according to Google Scholar, whereas the user manual of the stage of decision-making scale [50] has only been cited a little over 30 times and, when it is assessed, it is mostly done to include it as a covariate [66,67]. Consequently, the roles of these decisional stages seem to be much less understood. That said, it is assumed that individuals’ stages of decision-making influence their receptiveness to DAs and that people who are in active stages of deliberation would benefit the most from DAs, whereas people who either have not even begun to think about the decision or are unwilling to reconsider their decision may not benefit as much from a DA [50]. Interestingly, the largest group (approximately 40%) in our sample was at an early stage of decision-making, which is also reflected in the respondents’ average score for this variable (ie, 2.92, SD 1.4), which is slightly below active deliberation (ie, the stage in which individuals start weighing the different options). As such, it might be the case that *VISOR* (with or without the explicit VCM and advice) simply overwhelmed a big part of our sample. The relatively high decisional conflict scores in both groups seem to confirm this. Therefore, tailoring DAs to the stage of decision-making might be a promising approach to further limiting attrition. Although demographic variables seem to be the more obvious choice based on our data, the 1-item scale used to assess the stage of decision-making has the major advantage of being easy to use and not requiring participants to complete lengthy questionnaires. By tailoring DAs to the stage of decision-making (as opposed to other constructs or demographic characteristics), it would be possible to alleviate one of the core problems of most contemporary approaches to computer tailoring—that they often impose a significant burden on participants [68]. The fact that participants’ stage of decision-making had the most consistent effect on the outcomes among the included predictors only adds to this. For example, individuals in the initial stages could be offered interventions that are merely educational and supportive in nature, whereas individuals in active deliberation stages could be offered traditional DAs, and individuals in later stages could be offered support to implement their decision. To illustrate, individuals in an early decision-making stage could be offered a brief intervention aimed at increasing motivation to stop smoking or use evidence-based cessation assistance (similar to the 5 As [69]). Once they have reached a higher stage, they could then be provided with a traditional DA, such as *VISOR.* In other words, insights from behavior change could be used to further improve DAs for specific groups of decision makers [70].

### True Absence of an Effect

Owing to the lack of statistical power, we cannot confidently conclude that there truly was no effect of *VISOR*. However, interestingly, Sheridan et al [71] also tested the additional effect of an explicit VCM added to a DA aimed at a prevention-related decision (ie, heart disease prevention) and found no effect on outcomes such as decisional conflict or intention to reduce heart disease risk. However, most other studies tested DAs (and by extension explicit VCMs) in a treatment context (eg, between different surgeries [25]), with positive effects reported quite often. On the basis of this contradiction, we could deduce that prevention-related decisions are somewhat different and may not be affected by DAs in a manner similar to treatment decisions. Anecdotally, throughout the project in which *VISOR* was developed and tested, people not involved in the project often stated that prevention-related decisions (such as smoking cessation decisions) seemed easier to make than other decisions, such as treatment decisions. Although this conclusion is logical to a certain degree, as treatment decisions often involve much more imminent risks, such as death in the near future [25], we found no indications that the participants in this study regarded the decision among different cessation aids as easy. In fact, participants in both trial arms experienced decisional conflict that scored above the accepted cutoff value of 37.5 [51]. The decisional conflict scores observed in this study were also higher than those reported in the literature [72], which tend to focus on treatment decisions, indicating that prevention-related decisions are not necessarily easier to make than treatment decisions. In other words, there is no indication that prevention-related decisions are perceived differently from treatment decisions (at least not in terms of experienced difficulty), meaning that they can be assumed to respond similarly to intervention elements commonly included in DAs (such as VCMs). However, based on our data, it cannot be fully excluded that prevention-related decisions differ in factors other than experienced difficulty and therefore do not respond to explicit VCMs as expected. A more viable difference between prevention-related DAs and DAs focused on treatment decisions is that those focused on prevention are often used without the direct involvement of a health care professional [16,17]; that is, prevention-related DAs are more likely to be self-administered [73] than DAs focused on treatments. However, a recent systematic review and meta-analysis by Larsen et al [73] showed that self-administered DAs for colorectal cancer screening can also be beneficial in relation to prevention-related decisions, such as population screening, showcasing that this explanation (ie, lack of health care involvement) is unlikely. In other words, at this point, no convincing argument can be made about prevention-related decisions being different from other health care decisions. Therefore, they should respond similarly to the elements typically used in DAs. However, given the substantial amount of heterogeneity in the literature on DAs aimed at primary prevention [74], it may be worth investigating further whether prevention-related decisions really respond to DA elements, as one would expect based on the wider DA literature; for example, by replicating our work with a larger sample. In addition, more research could be conducted on perceived differences between prevention-related decisions and other health-related decisions (eg, treatment decisions); for example, by conducting in-depth interviews with participants who recently took both to discover perceived differences in their decision-making.

### Strengths and Limitations

The major strength of our study was that it was conducted in a *real-life* context, and we mainly recruited individuals who were interested in smoking cessation (as opposed to the hypothetical scenario often used to evaluate VCMs [25]). In addition, our sample was relatively representative of the Dutch population, except for women being overrepresented and participants with a low level of education being slightly underrepresented [75]. Thus, our findings might be largely generalizable to the smoking Dutch population with access to the internet. The fact that both groups received a DA also allowed us to blind participants, which strengthened the RCT, as participants were not biased in the sense that they knew to which intervention arm they were allocated and, thus, what the other group was offered. Finally, we focused our analyses on 3 outcomes of interest, thereby decreasing the risk of a type I error.

However, this study also has some limitations. Owing to our study design, we might have experienced a selection bias as we only invited people to complete the follow-ups if they had used the entire intervention. We tried to alleviate the influence of this selection bias by including variables associated with this selection (eg, age) as covariates in the analysis and imputation models. However, it is possible that more motivated smokers completed the intervention and therefore, for example, showed higher quit rates. In addition, we had to change the primary outcome and shorten the follow-up period to 6 months after baseline. Although unfortunate, we do not consider this to be a major issue as experts regard a follow-up period of 6 months to be sufficient, as most relapses occur relatively soon after the quit attempt [76-78]. In addition, the trial showed such large attrition that we were underpowered for most of our analyses. That said, the findings from the complete case analysis and those based on MI led to the same conclusions, making our findings more robust. It might also be a limitation that we relied on self-reported data. However, as previous research has shown that self-reported smoking status tends to be accurate [79] and because participants received the reward for study completion regardless of their smoking status, we are confident that this has had little to no influence on our findings. Finally, we were unable to separately test the effects of explicit VCM and computer-tailored advice. This would have required 3 trial arms and an even larger sample size and was therefore deemed unfeasible. It should also be recognized that digital, web-based DAs, by definition, exclude people who either do not have access to the internet at all or do not have the necessary digital literacy to use it. Although we consider this limitation to be small in the Dutch context, given the large number of Dutch households with internet access [80], it is certainly important to consider this to achieve full health equity. In the same vein, it is also important to consider other characteristics (eg, health literacy and financial resources to access smoking cessation assistance) that may influence the ability of end users to benefit from smoking cessation DAs. For example, the findings of our attrition analyses show that future projects should place a stronger emphasis on end users with less formal education. Finally, this study focused on direct behavioral and decisional effects only, as indirect effects were beyond the scope of this study. The same applies to cost-effectiveness and cost-utility. Investigating indirect effects (as hypothesized in the study protocol [28]), cost-effectiveness, and cost-utility might provide additional insights, in addition to the direct effects reported in this study.

### Conclusions

At this point, we cannot confidently recommend the inclusion of explicit VCMs and computer-tailored advice in smoking cessation DAs. In fact, these 2 elements might result in higher attrition rates during the use of DAs, thereby limiting their potential. However, our findings in relation to the primary and secondary outcomes might be influenced by a lack of statistical power; therefore, we advocate for the replication of our trial with a larger sample. Finally, researchers should emphasize user experience, especially use times, and experiment with (innovative) solutions to deal with a (too) high perceived user burden, such as the integration of *VISOR* within a counseling pathway or tailoring *VISOR* and other DAs to people’s decision-making stages. Finally, researchers should investigate how prevention-related decisions respond to DA elements. Given the strong initial interest in *VISOR* and the potential of smoking cessation DAs to combat tobacco-related mortality by inducing more smoking cessation attempts [15], future research should continue to find ways of optimizing smoking cessation DAs for future use.

## Appendix

Multimedia Appendix 1 Checklist for Reporting Results of Internet E-Surveys (CHERRIES).

Multimedia Appendix 2 Details on baseline measurements of demographic information and smoking behavior.

Multimedia Appendix 3 Variables included in the imputation model to test hypotheses 1a, 1b, 2a, and 2b.

Multimedia Appendix 4 Variables included in the imputation model to test hypothesis 3.

Multimedia Appendix 5 Baseline differences between participants who completed decision aid and those who did not.

Multimedia Appendix 6 Baseline differences between participants who completed time point 1 and the decision aid and those who did not complete time point 1.

Multimedia Appendix 7 Baseline differences between participants who completed time point 2 and the decision aid and those who did not complete time point 2.

Multimedia Appendix 8 Baseline differences between participants who completed time point 3 and the decision aid and those who did not complete time point 3.

Multimedia Appendix 9 Results of logistic regression for hypothesis 1b (worst-case scenario adjusted with the Revised Fagerström Test for Nicotine Dependence removed): smoking cessation after 6 months.

Multimedia Appendix 10 CONSORT-eHEALTH checklist (V 1.6.1).

## Abbreviations

CONSORT-EHEALTH: Consolidated Standards of Reporting Trials of Electronic and Mobile Health Applications and Online Telehealth
DA: decision aid
FTND-R: Revised Fagerström Test for Nicotine Dependence
IPDAS: International Patient Decision Aid Standards Collaboration
MI: multiple imputation
OR: odds ratio
OSF: open science framework
RCT: randomized controlled trial
SDT: self-determination theory
t=1: time point 1
t=2: time point 2
t=3: time point 3
VCM: value clarification method
