# Supplementary material for: Effects of an Explicit Value Clarification Method With Computer-Tailored Advice on the Effectiveness of a Web-Based Smoking Cessation Decision Aid: Findings From a Randomized Controlled Trial
Source: J Med Internet Res. 2022 Jul 15;24(7):e34246. doi: 10.2196/34246 (PMC9338418; doi:10.2196/34246)
Supplement: Multimedia Appendix 1 [file jmir_v24i7e34246_app1.docx]

**Multimedia Appendix 1.** Checklist for Reporting Results of Internet E-Surveys (CHERRIES)

| ***Item Category*** | ***Checklist Item*** | ***Explanation*** | ***Explanation related to our study*** |
| --- | --- | --- | --- |
| **Design** | Describe survey design | Describe target population, sample frame. Is the sample a convenience sample? (In “open” surveys this is most likely.) | Participants could be included if they were (1) currently smoking, (2) motivated to stop smoking within 6 months, (3) between 18–100 years old, (4) able to understand Dutch, and (5) had access to the internet and the necessary internet literacy (skills) to use the DA. Therefore, the sample frame consisted of virtually all adult smokers who spoke Dutch and had access to the internet. The sample was a convenience sample in the sense that anyone wishing to use the intervention could participate as long as they met the inclusion criteria. |
| **IRB (Institutional Review Board) approval and informed consent process** | IRB approval | Mention whether the study has been approved by an IRB. | The study did not fall under the scope of the Medical Research Involving Human Subjects Act as indicated by the Medical Ethics Committee Zuyderland, the Netherlands (16-N-227). |
|  | Informed consent | Describe the informed consent process. Where were the participants told the length of time of the survey, which data were stored and where and for how long, who the investigator was, and the purpose of the study? | Participants registered for the study via an online form, which included their provision of informed consent and the creation of an account. In general, the participants received an indication of the duration of the questionnaires and received the other information in the form of a privacy statement. |
|  | Data protection | If any personal information was collected or stored, describe what mechanisms were used to protect unauthorized access. | To ensure this, we initially chose to collect only personal information that was strictly necessary for our study or the intervention itself. In addition, only a very small group had access to this data. |
| **Development and pre-testing** | Development and testing | State how the survey was developed, including whether the usability and technical functionality of the electronic questionnaire had been tested before fielding the questionnaire. | The questionnaires used for the intervention described in this article were developed alongside the intervention. If available, we used previously validated measurements and measurements that were used in a Dutch context before, if possible, we used measurements that were used in self-administered online studies before. The questionnaires were pre-tested before they were employed in the study context, both by individuals involved in the project and by others. |
| **Recruitment process and description of the sample having access to the questionnaire** | Open survey versus closed survey | An "open survey" is a survey open for each visitor of a site, while a closed survey is only open to a sample which the investigator knows (password-protected survey). | The survey was open in the sense that anyone could participate as long as they met the inclusion criteria. However, participants had to create an account including a password. See also previous answers and our article for more information. |
|  | Contact mode | Indicate whether or not the initial contact with the potential participants was made on the Internet. (Investigators may also send out questionnaires by mail and allow for Web-based data entry.) | Most of the first contacts were indeed online, because we mainly recruited via social media. See *Study Population* for in-depth information. That said, at no time did the participants and the authors interact directly, online, or otherwise. |
|  | Advertising the survey | How/where was the survey announced or advertised? Some examples are offline media (newspapers), or online (mailing lists – If yes, which ones?) or banner ads (Where were these banner ads posted and what did they look like?). It is important to know the wording of the announcement as it will heavily influence who chooses to participate. Ideally the survey announcement should be published as an appendix. | Participants were mainly recruited online to reflect the online nature of the intervention and the entire trial was web-based (ie, there were no offline contacts). Recruitment took mainly place by making use of paid social media advertisements and unpaid social media posts on project accounts that were also shared on the team members' accounts, their respective institutions, and other relevant organizations within the Netherlands. Additionally, the intervention was also featured in regional media (eg, a newspaper interview) and we used a project website with a direct access point to the intervention via a clickable button. The project's social media accounts are still online, and so are all recruitment posts–see for example Instagram: <https://www.instagram.com/keuzehulp_visor>. |
| **Survey administration** | Web/E-mail | State the type of e-survey (eg, one posted on a Web site, or one sent out through e-mail). If it is an e-mail survey, were the responses entered manually into a database, or was there an automatic method for capturing responses? | The survey was embedded in the same web application as the intervention itself. |
|  | Context | Describe the Web site (for mailing list/newsgroup) in which the survey was posted. What is the Web site about, who is visiting it, what are visitors normally looking for? Discuss to what degree the content of the Web site could pre-select the sample or influence the results. For example, a survey about vaccination on an anti-immunization Web site will have different results from a Web survey conducted on a government Web site | See the checklist item *Advertising the survey*. |
|  | Mandatory/voluntary | Was it a mandatory survey to be filled in by every visitor who wanted to enter the Web site, or was it a voluntary survey? | Participants that wanted to use the intervention also had to fill in the baseline questionnaire. All other questionnaires were voluntary. |
|  | Incentives | Were any incentives offered (eg, monetary, prizes, or non-monetary incentives such as an offer to provide the survey results)? | When participants completed the last questionnaire after 6 months, they received € 10, which approximately equates to US $ 12.17 at the time of writing. Also, participants gained access to an intervention when they filled in the baseline questionnaire. |
|  | Time/Date | In what timeframe were the data collected? | Data was collected between January 2020 and August 2021. |
|  | Randomization of items or questionnaires | To prevent biases items can be randomized or alternated. | Items were neither randomized nor alternated. |
|  | Adaptive questioning | Use adaptive questioning (certain items, or only conditionally displayed based on responses to other items) to reduce number and complexity of the questions. | Items were automatically adapted depending on the respective answers, eg, if the participants stated that they have never attempted to cease smoking, no follow-up questions were posed to them. This information can be found in the protocol article: <https://doi.org/10.2196/21772>. |
|  | Number of Items | What was the number of questionnaire items per page? The number of items is an important factor for the completion rate. | The baseline questionnaire (t=0) consisted of 22–94 items, t=1 of 53–56 items, t=2 of 14–21 items, and t=3 of 16–88 items. This information can be found in the protocol article: <https://doi.org/10.2196/21772>. The number of items per page varied per page. |
|  | Number of screens (pages) | Over how many pages was the questionnaire distributed? The number of items is an important factor for the completion rate. | The baseline questionnaire (t=0) consisted of 23 pages, t=1 of 13 pages, t=2 of 8 pages, and t=3 of 21 pages. Note that some of these pages were skipped if all items on that page were skipped, see also the previous 2 checklist items. |
|  | Completeness check | It is technically possible to do consistency or completeness checks before the questionnaire is submitted. Was this done, and if “yes”, how (usually JAVAScript)? An alternative is to check for completeness after the questionnaire has been submitted (and highlight mandatory items). If this has been done, it should be reported. All items should provide a non-response option such as “not applicable” or “rather not say”, and selection of one response option should be enforced. | Because we used forced-choice questions, all completed questionnaires were complete. |
|  | Review step | State whether respondents were able to review and change their answers (eg, through a Back button or a Review step which displays a summary of the responses and asks the respondents if they are correct). | Participants could not change their answers once they moved to the next page. |
| **Response rates** | Unique site visitor | If you provide view rates or participation rates, you need to define how you determined a unique visitor. There are different techniques available, based on IP addresses or cookies or both. | We did not register unique site visitors in the sense that everyone who visited the website hosting the intervention and questionnaires was registered. |
|  | View rate (Ratio of unique survey visitors/unique site visitors) | Requires counting unique visitors to the first page of the survey, divided by the number of unique site visitors (not page views!). It is not unusual to have view rates of less than 0.1 % if the survey is voluntary. | Information not available, see *Unique site visitor*. |
|  | Participation rate (Ratio of unique visitors who agreed to participate/unique first survey page visitors) | Count the unique number of people who filled in the first survey page (or agreed to participate, for example by checking a checkbox), divided by visitors who visit the first page of the survey (or the informed consents page, if present). This can also be called “recruitment” rate. | Information not available, see *Unique site visitor*. |
|  | Completion rate (Ratio of users who finished the survey/users who agreed to participate) | The number of people submitting the last questionnaire page, divided by the number of people who agreed to participate (or submitted the first survey page). This is only relevant if there is a separate “informed consent” page or if the survey goes over several pages. This is a measure for attrition. Note that “completion” can involve leaving questionnaire items blank. This is not a measure for how completely questionnaires were filled in. (If you need a measure for this, use the word “completeness rate”.) | Depending on the measurement points: Baseline/t=0: 1164/2375 (49.01%), t=1: 276/2375 (11.62%), t=2: 97/2375 (4.08%), t=3: 103/2375 (4.34%). However, it should be stressed that only participants that completed the intervention between t=0 and t=1 were invited to follow-up. See the article itself for more information. |
| **Preventing multiple entries from the same individual** | Cookies used | Indicate whether cookies were used to assign a unique user identifier to each client computer. If so, mention the page on which the cookie was set and read, and how long the cookie was valid. Were duplicate entries avoided by preventing users access to the survey twice; or were duplicate database entries having the same user ID eliminated before analysis? In the latter case, which entries were kept for analysis (eg, the first entry or the most recent)? | Not applicable |
|  | IP check | Indicate whether the IP address of the client computer was used to identify potential duplicate entries from the same user. If so, mention the period of time for which no two entries from the same IP address were allowed (eg, 24 hours). Were duplicate entries avoided by preventing users with the same IP address access to the survey twice; or were duplicate database entries having the same IP address within a given period of time eliminated before analysis? If the latter, which entries were kept for analysis (eg, the first entry or the most recent)? | Not applicable |
|  | Log file analysis | Indicate whether other techniques to analyze the log file for identification of multiple entries were used. If so, please describe. | Not applicable |
|  | Registration | In “closed” (non-open) surveys, users need to login first and it is easier to prevent duplicate entries from the same user. Describe how this was done. For example, was the survey never displayed a second time once the user had filled it in, or was the username stored together with the survey results and later eliminated? If the latter, which entries were kept for analysis (eg, the first entry or the most recent)? | Participants had to register and create an account in the process. Once participants filled in one of the questionnaires (eg, t=1) with their respective accounts, they were never shown those questionnaires (eg, t=1) again. |
| **Analysis** | Handling of incomplete questionnaires | Were only completed questionnaires analyzed? Were questionnaires which terminated early (where, for example, users did not go through all questionnaire pages) also analyzed? | For effect analyses we only used the data of users that used the entire intervention. However, we used multiple approaches to handle missingness after the intervention (if applicable): (1) Complete cases only, (2) worst-case scenario (dropout respondents were considered to still smoke, ie, penalized imputation), (3) multiple imputations (MI) using the *mice* package. See *Data Analysis* for more information. |
|  | Questionnaires submitted with an atypical timestamp | Some investigators may measure the time people needed to fill in a questionnaire and exclude questionnaires that were submitted too soon. Specify the timeframe that was used as a cut-off point and describe how this point was determined. | Not applicable |
|  | Statistical correction | Indicate whether any methods such as weighting of items or propensity scores have been used to adjust for the non-representative sample; if so, please describe the methods. | Not applicable |
