# Supplementary material for: Effects of an Explicit Value Clarification Method With Computer-Tailored Advice on the Effectiveness of a Web-Based Smoking Cessation Decision Aid: Findings From a Randomized Controlled Trial
Source: J Med Internet Res. 2022 Jul 15;24(7):e34246. doi: 10.2196/34246 (PMC9338418; doi:10.2196/34246)
Supplement: Multimedia Appendix 2 [file jmir_v24i7e34246_app2.docx]

**Multimedia Appendix 2.** Details baseline measurements demographic information and smoking behavior

| **Measurements** | **Answer categories** | **Other information** |
| --- | --- | --- |
| **Demographic information** | | |
| Age | 1 = 18-23  2 = 24-29  3 = 30-100 | Initially collected continuously but was recoded into 3 categories to comply with the privacy regulations of the University of Amsterdam. |
| Gender | 0 = woman  1 = man  2 = not listed/non-binary  3 = prefer not to say | N/A^a^ |
| Education | 0 = low  1 = medium  2 = high | Was originally collected as the highest educational qualification achieved to be recoded into three categories based on Statistics Netherlands (the Dutch Central Agency for Statistics) [1]: 0 = low (including either only or no primary education, and lower vocational education), 1 = medium (including general secondary education, secondary vocational education, and secondary general education) and 2 = high (including higher professional education and university education). |
| **Smoking behavior** | | |
| Tobacco products used | 0 = cigarette  1 = hand-rolled cigarette  2 = e-cigarette  3 = pipe  4 = other products | Due to a substantial number of participants indicating that they used cannabis products (eg, joints) and cigars, we created variables reflecting this. If participants only indicated those options under "4 = other products", we manually removed "4 = other products" from their data to prevent double counting. |
| Amount of tobacco consumption per product per day | Continuously, except for e-cigarettes which was measured categorically:  1 = less than monthly  2 = less than weekly, but at least once per month  3 = less than daily, but at least once per week  4 = daily, but not multiple times per day  5 = multiple times per day | N/A^a^ |
| Past cessation attempts | 0 = yes  1 = no | N/A^a^ |
| Amount of past cessation attempts | Continuously | N/A^a^ |
| Cessation assistance utilization in the past six month | 0 = yes  1 = no | N/A^a^ |
| Cessation assistance utilization in the past six month (if participants indicated that they have utilized cessation assistance) | 1 = varenicline  2 = bupropion  3 = nortriptyline  4 = nicotine patches  5 = nicotine gums  6 = nicotine lozenge  7 = nicotine mouth spray  8 = nicotine inhaler  9 = face-to-face counseling 10 = counseling over the phone  11 = group coaching  12 = eHealth  13 = something else  14 = cessation without assistance | A substantial group of people indicated similar options under "13 = something else" (ie, cytisine and options not clearly described but with a high chance of being effective (eg, general practitioners)). We, therefore, also chose to create variables for those two options (again, we manually removed "13 = something else", if those were the only options that people used). Both options were regarded as being evidence-based. However, it should be said that cytisine is not legally available at this point in the Netherlands–nevertheless, we chose to regard it as being evidence-based as it has been shown to be effective in individual studies [2] and meta-analyses [3].  During data cleaning, we also saw that a very small group of people indicated to only have made 1 cessation attempt, but also indicated to have stopped without any assistance *and* with assistance–which is logically impossible. We, therefore, removed "14 = cessation without assistance" from their entries.  Ultimately, this information was recoded (eg, 0 = did not use evidence-based cessation assistance, 1 = used evidence-based cessation assistance). |

**Note.** ^a^N/A: not applicable.

1. Centraal Bureau voor de Statistiek. Opleidingsniveau [Internet]. [cited 2021 Apr 21]. Available from: https://www.cbs.nl/nl-nl/nieuws/2019/33/verschil-levensverwachting-hoog-en-laagopgeleid-groeit/opleidingsniveau

2. West R, Zatonski W, Cedzynska M, Lewandowska D, Pazik J, Aveyard P, Stapleton J. Placebo-Controlled Trial of Cytisine for Smoking Cessation. N Engl J Med Massachusetts Medical Society; 2011 Sep 29;365:1193–1200. [doi: 10.1056/NEJMoa1102035]

3. Hajek P, McRobbie H, Myers K. Efficacy of cytisine in helping smokers quit: systematic review and meta-analysis. Thorax 2013 Nov 1;68(11):1037–1042. [doi: 10.1136/thoraxjnl-2012-203035]
