# Supplementary material for: Effects of an Explicit Value Clarification Method With Computer-Tailored Advice on the Effectiveness of a Web-Based Smoking Cessation Decision Aid: Findings From a Randomized Controlled Trial
Source: J Med Internet Res. 2022 Jul 15;24(7):e34246. doi: 10.2196/34246 (PMC9338418; doi:10.2196/34246)
Supplement: Multimedia Appendix 3 [file jmir_v24i7e34246_app3.docx]

**Multimedia Appendix 3.** Variables included in the imputation model to test H_1a/b_ and H_2a/b_

| **Variables that were also part of the analysis model** | Group allocation, age, gender, education, Revised Fagerström Test for Nicotine Dependence (FTND-R), and stage of decision making |
| --- | --- |
| **Other additional variables that were also related to dropout** | Whether the invite for t=2 was sent (only for H_1a_ and H_2a_), whether participants only shared the answer to the primary outcome with the research team (only for H_1b_ and H_2b_), and whether the date for t=3 had been adjusted (only for H_1b_ and H_2b_) |
| **Auxiliary variables:** | Evidence-based cessation assistance utilization in the past 6 months (at baseline) |
