# Supplementary material for: Effects of an Explicit Value Clarification Method With Computer-Tailored Advice on the Effectiveness of a Web-Based Smoking Cessation Decision Aid: Findings From a Randomized Controlled Trial
Source: J Med Internet Res. 2022 Jul 15;24(7):e34246. doi: 10.2196/34246 (PMC9338418; doi:10.2196/34246)
Supplement: Multimedia Appendix 4 [file jmir_v24i7e34246_app4.docx]

**Multimedia Appendix 4.** Variables included in the imputation model to test H_3_

| **Variables that were also part of the analysis model** | Group allocation, age, gender, education, and stage of decision making |
| --- | --- |
| **Other additional variables that were also related to dropout** | N/A^a^ |
| **Auxiliary variables:** | N/A^a^ |

**Note.** ^a^N/A: not applicable.
