# Supplementary material for: Effects of an Explicit Value Clarification Method With Computer-Tailored Advice on the Effectiveness of a Web-Based Smoking Cessation Decision Aid: Findings From a Randomized Controlled Trial
Source: J Med Internet Res. 2022 Jul 15;24(7):e34246. doi: 10.2196/34246 (PMC9338418; doi:10.2196/34246)
Supplement: Multimedia Appendix 5 [file jmir_v24i7e34246_app5.docx]

**Multimedia Appendix 5.** Baseline differences between participants who completed the DA with those who did not

| **Participant characteristics** | **Entire sample**  (N = 1164) | **DA completed**  (n = 599) | **DA not completed**  (n = 565) | ***P*-value** |
| --- | --- | --- | --- | --- |
| **Group allocation** |  |  |  | < .001 |
| Intervention | 599 (51.5%) | 275 (45.9%) | 324 (57.3%) |  |
| Control | 565 (48.5%) | 324 (54.1%) | 241 (42.7%) |  |
| **Gender** |  |  |  | .32^a^ |
| Women, n (%) | 738 (63.4%) | 388 (64.8%) | 350 (61.9%) |  |
| Men, n (%) | 424 (36.4%) | 210 (35.1%) | 214 (37.9%) |  |
| Non-binary, n (%) | 1 (0.1%) | 1 (0.2%) | 0 (0.0%) |  |
| Prefers not to say, n (%) | 1 (0.1%) | 0 (0.0%) | 1 (0.2%) |  |
| **Age** |  |  |  | < .001 |
| 18–23, n (%) | 326 (28.0%) | 107 (17.9%) | 219 (38.8%) |  |
| 24–29, n (%) | 155 (13.3%) | 69 (11.5%) | 86 (15.2%) |  |
| 30–100, n (%) | 683 (58.7%) | 423 (70.6%) | 260 (46.0%) |  |
| **Education** |  |  |  | .002 |
| Low, n (%) | 151 (13.0%) | 68 (11.4%) | 83 (14.7%) |  |
| Medium, n (%) | 661 (56.8%) | 323 (53.9%) | 338 (59.8%) |  |
| High, n (%) | 352 (30.2%) | 208 (34.7%) | 144 (25.5%) |  |
| **Tobacco products**^1^ |  |  |  |  |
| Cigarettes, n (%) | 1144 (98.3%) | 587 (98.0%) | 557 (98.6%) | .44 |
| E-cigarettes^2^, n (%) | 56 (4.8%) | 27 (4.5%) | 29 (5.1%) | .62 |
| Pipe, n (%) | 6 (0.5%) | 3 (0.5%) | 3 (0.5%) | < .99 |
| Cannabis, n (%) | 42 (3.6%) | 19 (3.2%) | 23 (4.1%) | .41 |
| Cigar, n (%) | 17 (1.5%) | 10 (1.7%) | 7 (1.2%) | .54 |
| Other, n (%) | 16 (1.4%) | 4 (0.7%) | 12 (2.1%) | .03 |
| **Tobacco consumption** |  |  |  |  |
| Total without e-cigarettes (daily), median | 15 | 15 | 15 | .17 |
| E-cigarettes only^3^ |  |  |  | .11 |
| *Less than monthly, n (%)* | 6 (10.7%) | 0 (0%) | 6 (20.7%) |  |
| *Less than weekly, but at least once per month, n (%)* | 10 (17.9%) | 6 (22.2%) | 4 (13.8%) |  |
| *Less than daily, but at least once per week, n (%)* | 12 (21.4%) | 6 (22.2%) | 6 (20.7%) |  |
| *Daily, but not multiple times, n (%)* | 4 (7.1%) | 3 (11.1%) | 1 (3.4%) |  |
| *Multiple times per day, n (%)* | 24 (42.9%) | 12 (44.4%) | 12 (41.4%) |  |
| **Smoking cessation behavior** |  |  |  |  |
| Ever smoking cessation attempt, n (%) | 1032 (88.7%) | 539 (90.0%) | 493 (87.3%) | .14 |
| Amount of smoking cessation attempts (lasting 24h), median^4^ | 3 | 3 | 3 | .02 |
| Cessation assistance utilization in the past 6 months (%) |  |  |  |  |
| *Evidence-based*^5^*, n (%)* | 169 (14.5%) | 89 (14.9%) | 80 (14.2%) | .74 |
| *Non-evidence-based*^5^*, n (%)* | 23 (2.0%) | 16 (2.7%) | 7 (1.2%) | .08 |
| **Stage of decision making** |  |  |  |  |
| Has not yet started to think about the choice, n (%) | 185 (15.9%) | 75 (12.5%) | 110 (19.5%) |  |
| Has not started thinking about the choice yet, but wants to do it, n (%) | 288 (24.7%) | 147 (24.5%) | 141 (25.0%) |  |
| Is currently weighing the different options, n (%) | 404 (34.7%) | 220 (36.7%) | 184 (32.6%) |  |
| Almost chose an option, n (%) | 87 (7.5%) | 45 (7.5%) | 42 (7.4%) |  |
| Already made a decision, but is still ready to consider, n (%) | 124 (10.7%) | 69 (11.5%) | 55 (9.7%) |  |
| Has already made up their mind and will probably not change their mind, n (%) | 76 (6.5%) | 43 (7.2%) | 33 (5.8%) |  |
| Median | 3 | 3 | 3 | .004 |
| **FTND-R**, median | 7 | 7 | 7 | .42 |

**Note.** DA = decision aid; FTND-R = Revised Fagerström Test for Nicotine Dependence; ^a^excluding the groups 'non-binary' and 'Prefers not to say'; ^1^selecting multiple products was possible; ^2^all dual users, ^3^percentages refer to e-cigarette users only, ^4^excluding extreme outliers ≥1000 and participants that never attempted to stop smoking before, ^5^at least one, can be multiple; percentages exceeding 100% are due to rounding.
