# Supplementary material for: Effects of an Explicit Value Clarification Method With Computer-Tailored Advice on the Effectiveness of a Web-Based Smoking Cessation Decision Aid: Findings From a Randomized Controlled Trial
Source: J Med Internet Res. 2022 Jul 15;24(7):e34246. doi: 10.2196/34246 (PMC9338418; doi:10.2196/34246)
Supplement: Multimedia Appendix 6 [file jmir_v24i7e34246_app6.docx]

**Multimedia Appendix 6.** Baseline differences between participants who completed t=1 *and* the DA with those that did not complete t=1

| **Participant characteristics** | **DA completed**  (n = 599) | **T = 1 completed**  (n = 276) | **T = 1 not completed**  (n = 323) | ***P*-value** |
| --- | --- | --- | --- | --- |
| **Group allocation** |  |  |  | .01 |
| Intervention | 275 (45.9%) | 143 (51.8%) | 132 (40.9%) |  |
| Control | 324 (54.1%) | 133 (48.2%) | 191 (59.1%) |  |
| **Gender** |  |  |  | .02^a^ |
| Women, n (%) | 388 (64.8%) | 165 (59.8%) | 223 (69.0%) |  |
| Men, n (%) | 210 (35.1%) | 111 (40.2%) | 99 (30.7%) |  |
| Non-binary, n (%) | 1 (0.2%) | 0 (0.0%) | 1 (0.3%) |  |
| Prefers not to say, n (%) | 0 (0.0%) | 0 (0.0%) | 0 (0.0%) |  |
| **Age** |  |  |  | .10 |
| 18–23, n (%) | 107 (17.9%) | 45 (16.3%) | 62 (19.2%) |  |
| 24–29, n (%) | 69 (11.5%) | 25 (9.1%) | 44 (13.6%) |  |
| 30–100, n (%) | 423 (70.6%) | 206 (74.6%) | 217 (67.2%) |  |
| **Education** |  |  |  | .07 |
| Low, n (%) | 68 (11.4%) | 33 (12.0%) | 35 (10.8%) |  |
| Medium, n (%) | 323 (53.9%) | 135 (48.9%) | 188 (58.2%) |  |
| High, n (%) | 208 (34.7%) | 108 (39.1%) | 100 (31.0%) |  |
| **Tobacco products**^1^ |  |  |  |  |
| Cigarettes, n (%) | 587 (98.0%) | 269 (97.5%) | 318 (98.5%) | .39 |
| E-cigarettes^2^, n (%) | 27 (4.5%) | 12 (4.3%) | 15 (4.6%) | .86 |
| Pipe, n (%) | 3 (0.5%) | 2 (0.7%) | 1 (0.3%) | .60 |
| Cannabis, n (%) | 19 (3.2%) | 6 (2.2%) | 13 (4.0%) | .20 |
| Cigar, n (%) | 10 (1.7%) | 5 (1.8%) | 5 (1.5%) | < .99 |
| Other, n (%) | 4 (0.7%) | 1 (0.4%) | 3 (0.9%) | .63 |
| **Tobacco consumption** |  |  |  |  |
| Total without e-cigarettes (daily), median | 15 | 15 | 15 | .71 |
| E-cigarettes only^3^ |  |  |  | .95 |
| *Less than monthly, n (%)* | 0 (0%) | 0 (0%) | 0 (0%) |  |
| *Less than weekly, but at least once per month, n (%)* | 6 (22.2%) | 3 (25.0%) | 3 (20.0%) |  |
| *Less than daily, but at least once per week, n (%)* | 6 (22.2%) | 2 (16.7%) | 4 (26.7%) |  |
| *Daily, but not multiple times, n (%)* | 3 (11.1%) | 1 (8.3%) | 2 (13.3%) |  |
| *Multiple times per day, n (%)* | 12 (44.4%) | 6 (50.0%) | 6 (40.0%) |  |
| **Smoking cessation behavior** |  |  |  |  |
| Ever smoking cessation attempt, n (%) | 539 (90.0%) | 244 (88.4%) | 295 (91.3%) | .23 |
| Amount of smoking cessation attempts (lasting 24h), median^4^ | 3 | 3 | 3 | .68 |
| Cessation assistance utilization in the past 6 months (%) |  |  |  |  |
| *Evidence-based*^5^*, n (%)* | 89 (14.9%) | 45 (16.3%) | 44 (13.6%) | .36 |
| *Non-evidence-based*^5^*, n (%)* | 16 (2.7%) | 8 (2.9%) | 8 (2.5%) | .75 |
| **Stage of decision making** |  |  |  |  |
| Has not yet started to think about the choice, n (%) | 75 (12.5%) | 24 (8.7%) | 51 (15.8%) |  |
| Has not started thinking about the choice yet, but wants to do it, n (%) | 147 (24.5%) | 66 (23.9%) | 81 (25.1%) |  |
| Is currently weighing the different options, n (%) | 220 (36.7%) | 97 (35.1%) | 123 (38.1%) |  |
| Almost chose an option, n (%) | 45 (7.5%) | 24 (8.7%) | 21 (6.5%) |  |
| Already made a decision, but is still ready to consider, n (%) | 69 (11.5%) | 41 (14.9%) | 28 (8.7%) |  |
| Has already made up their mind and will probably not change their mind, n (%) | 43 (7.2%) | 24 (8.7%) | 19 (5.9%) |  |
| Median | 3 | 3 | 3 | .001 |
| **FTND-R**, median | 7 | 7 | 7 | .5 |

**Note.** DA = decision aid; FTND-R = Revised Fagerström Test for Nicotine Dependence; ^a^excluding the groups 'non-binary' and 'Prefers not to say'; ^1^selecting multiple products was possible; ^2^all dual users, ^3^percentages refer to e-cigarette users only, ^4^excluding extreme outliers ≥1000 and participants that never attempted to stop smoking before, ^5^at least one, can be multiple; percentages exceeding 100% are due to rounding.
