# Supplementary material for: Effects of an Explicit Value Clarification Method With Computer-Tailored Advice on the Effectiveness of a Web-Based Smoking Cessation Decision Aid: Findings From a Randomized Controlled Trial
Source: J Med Internet Res. 2022 Jul 15;24(7):e34246. doi: 10.2196/34246 (PMC9338418; doi:10.2196/34246)
Supplement: Multimedia Appendix 7 [file jmir_v24i7e34246_app7.docx]

**Multimedia Appendix 7.** Baseline differences between participants who completed t=2 *and* the DA with those that did not complete t=2

| **Participant characteristics** | **DA completed**  (n = 599) | **T = 2 completed**  (n = 97) | **T = 2 not completed**  (n = 502) | ***P*-value** |
| --- | --- | --- | --- | --- |
| **Group allocation** |  |  |  | .04 |
| Intervention | 275 (45.9%) | 54 (55.7%) | 221 (44.0%) |  |
| Control | 324 (54.1%) | 43 (44.3%) | 281 (56.0%) |  |
| **Gender** |  |  |  | .99^a^ |
| Women, n (%) | 388 (64.8%) | 63 (64.9%) | 325 (64.7%) |  |
| Men, n (%) | 210 (35.1%) | 34 (35.1%) | 176 (35.1%) |  |
| Non-binary, n (%) | 1 (0.2%) | 0 (0.0%) | 1 (0.2%) |  |
| Prefers not to say, n (%) | 0 (0.0%) | 0 (0.0%) | 0 (0.0%) |  |
| **Age** |  |  |  | .25 |
| 18–23, n (%) | 107 (17.9%) | 21 (21.6%) | 86 (17.1%) |  |
| 24–29, n (%) | 69 (11.5%) | 7 (7.2%) | 62 (12.4%) |  |
| 30–100, n (%) | 423 (70.6%) | 69 (71.1%) | 354 (70.5%) |  |
| **Education** |  |  |  | .20 |
| Low, n (%) | 68 (11.4%) | 6 (6.2%) | 62 (12.4%) |  |
| Medium, n (%) | 323 (53.9%) | 54 (55.7%) | 269 (53.6%) |  |
| High, n (%) | 208 (34.7%) | 37 (38.1%) | 171 (34.1%) |  |
| **Tobacco products**^1^ |  |  |  |  |
| Cigarettes, n (%) | 587 (98.0%) | 94 (96.9%) | 493 (98.2%) | .42 |
| E-cigarettes^2^, n (%) | 27 (4.5%) | 5 (5.2%) | 22 (4.4%) | .79 |
| Pipe, n (%) | 3 (0.5%) | 1 (1.0%) | 2 (0.4%) | .41 |
| Cannabis, n (%) | 19 (3.2%) | 2 (2.1%) | 17 (3.4%) | .75 |
| Cigar, n (%) | 10 (1.7%) | 2 (2.1%) | 8 (1.6%) | .67 |
| Other, n (%) | 4 (0.7%) | 1 (0.1%) | 3 (0.6%) | .51 |
| **Tobacco consumption** |  |  |  |  |
| Total without e-cigarettes (daily), median | 15 | 15 | 15 | .44 |
| E-cigarettes only^3^ |  |  |  | .41 |
| *Less than monthly, n (%)* | 0 (0%) | 0 (0%) | 0 (0%) |  |
| *Less than weekly, but at least once per month, n (%)* | 6 (22.2%) | 2 (40.0%) | 4 (18.2%) |  |
| *Less than daily, but at least once per week, n (%)* | 6 (22.2%) | 0 (0%) | 6 (27.3%) |  |
| *Daily, but not multiple times, n (%)* | 3 (11.1%) | 1 (20.0%) | 2 (9.1%) |  |
| *Multiple times per day, n (%)* | 12 (44.4%) | 2 (40.0%) | 10 (45.5%) |  |
| **Smoking cessation behavior** |  |  |  |  |
| Ever smoking cessation attempt, n (%) | 539 (90.0%) | 83 (85.6%) | 456 (90.8%) | .11 |
| Amount of smoking cessation attempts (lasting 24h), median^4^ | 3 | 3 | 3 | .29 |
| Cessation assistance utilization in the past 6 months (%) |  |  |  |  |
| *Evidence-based*^5^*, n (%)* | 89 (14.9%) | 14 (14.4%) | 75 (14.9%) | .90 |
| *Non-evidence-based*^5^*, n (%)* | 16 (2.7%) | 1 (1.0%) | 15 (3.0%) | .49 |
| **Stage of decision making** |  |  |  |  |
| Has not yet started to think about the choice, n (%) | 75 (12.5%) | 8 (8.2%) | 67 (13.3%) |  |
| Has not started thinking about the choice yet, but wants to do it, n (%) | 147 (24.5%) | 18 (18.6%) | 129 (25.7%) |  |
| Is currently weighing the different options, n (%) | 220 (36.7%) | 40 (41.2%) | 180 (35.9%) |  |
| Almost chose an option, n (%) | 45 (7.5%) | 11 (11.3%) | 34 (6.8%) |  |
| Already made a decision, but is still ready to consider, n (%) | 69 (11.5%) | 12 (12.4%) | 57 (11.4%) |  |
| Has already made up their mind and will probably not change their mind, n (%) | 43 (7.2%) | 8 (8.2%) | 35 (7.0%) |  |
| Median | 3 | 3 | 3 | .03 |
| **FTND-R**, median | 7 | 7 | 7 | .22 |

**Note.** DA = decision aid; FTND-R = Revised Fagerström Test for Nicotine Dependence; ^a^excluding the groups 'non-binary' and 'Prefers not to say'; ^1^selecting multiple products was possible; ^2^all dual users, ^3^percentages refer to e-cigarette users only, ^4^excluding extreme outliers ≥1000 and participants that never attempted to stop smoking before, ^5^at least one, can be multiple; percentages exceeding 100% are due to rounding.
