# Supplementary material for: Effects of an Explicit Value Clarification Method With Computer-Tailored Advice on the Effectiveness of a Web-Based Smoking Cessation Decision Aid: Findings From a Randomized Controlled Trial
Source: J Med Internet Res. 2022 Jul 15;24(7):e34246. doi: 10.2196/34246 (PMC9338418; doi:10.2196/34246)
Supplement: Multimedia Appendix 8 [file jmir_v24i7e34246_app8.docx]

**Multimedia Appendix 8.** Baseline differences between participants who completed t=3 *and* the DA with those that did not complete t=3

| **Participant characteristics** | **DA completed**  (n = 599) | **T = 3 completed**  (n = 103) | **T = 3 not completed**  (n = 496) | ***P*-value** |
| --- | --- | --- | --- | --- |
| **Group allocation** |  |  |  | .06 |
| Intervention | 275 (45.9%) | 56 (54.4%) | 219 (44.2%) |  |
| Control | 324 (54.1%) | 47 (45.6%) | 277 (55.8%) |  |
| **Gender** |  |  |  | .52^a^ |
| Women, n (%) | 388 (64.8%) | 64 (62.1%) | 324 (65.3%) |  |
| Men, n (%) | 210 (35.1%) | 39 (37.9%) | 171 (34.5%) |  |
| Non-binary, n (%) | 1 (0.2%) | 0 (0.0%) | 1 (0.2%) |  |
| Prefers not to say, n (%) | 0 (0.0%) | 0 (0.0%) | 0 (0.0%) |  |
| **Age** |  |  |  | .67 |
| 18–23, n (%) | 107 (17.9%) | 21 (20.4%) | 86 (17.3%) |  |
| 24–29, n (%) | 69 (11.5%) | 10 (9.7%) | 59 (11.9%) |  |
| 30–100, n (%) | 423 (70.6%) | 72 (69.9%) | 351 (70.8%) |  |
| **Education** |  |  |  | .64 |
| Low, n (%) | 68 (11.4%) | 9 (8.7%) | 59 (11.9%) |  |
| Medium, n (%) | 323 (53.9%) | 58 (56.3%) | 265 (53.4%) |  |
| High, n (%) | 208 (34.7%) | 36 (35.0%) | 172 (34.7%) |  |
| **Tobacco products**^1^ |  |  |  |  |
| Cigarettes, n (%) | 587 (98.0%) | 101 (98.1%) | 486 (98.0%) | < .99 |
| E-cigarettes^2^, n (%) | 27 (4.5%) | 5 (4.9%) | 22 (4.4%) | .80 |
| Pipe, n (%) | 3 (0.5%) | 1 (1.0%) | 2 (0.4%) | .43 |
| Cannabis, n (%) | 19 (3.2%) | 2 (1.9%) | 17 (3.4%) | .76 |
| Cigar, n (%) | 10 (1.7%) | 1 (1.0%) | 9 (1.8%) | < .99 |
| Other, n (%) | 4 (0.7%) | 0 (0.0%) | 4 (0.8%) | < .99 |
| **Tobacco consumption** |  |  |  |  |
| Total without e-cigarettes (daily), median | 15 | 15 | 15 | .57 |
| E-cigarettes only^3^ |  |  |  | .67 |
| *Less than monthly, n (%)* | 0 (0.0%) | 0 (0.0%) | 0 (0.0%) |  |
| *Less than weekly, but at least once per month, n (%)* | 6 (22.2%) | 1 (20.0%) | 5 (22.7%) |  |
| *Less than daily, but at least once per week, n (%)* | 6 (22.2%) | 0 (0.0%) | 6 (27.3%) |  |
| *Daily, but not multiple times, n (%)* | 3 (11.1%) | 1 (20.0%) | 2 (9.1%) |  |
| *Multiple times per day, n (%)* | 12 (44.4%) | 3 (60.0%) | 9 (40.9%) |  |
| **Smoking cessation behavior** |  |  |  |  |
| Ever smoking cessation attempt, n (%) | 539 (90.0%) | 91 (88.3%) | 448 (90.3%) | .54 |
| Amount of smoking cessation attempts (lasting 24h), median^4^ | 3 | 3 | 3 | .61 |
| Cessation assistance utilization in the past 6 months (%) |  |  |  |  |
| *Evidence-based*^5^*, n (%)* | 89 (14.9%) | 17 (16.5%) | 72 (14.5%) | .61 |
| *Non-evidence-based*^5^*, n (%)* | 16 (2.7%) | 2 (1.9%) | 14 (2.8%) | > .99 |
| **Stage of decision making** |  |  |  |  |
| Has not yet started to think about the choice, n (%) | 75 (12.5%) | 7 (6.8%) | 68 (13.7%) |  |
| Has not started thinking about the choice yet, but wants to do it, n (%) | 147 (24.5%) | 21 (20.4%) | 126 (25.4%) |  |
| Is currently weighing the different options, n (%) | 220 (36.7%) | 40 (38.85) | 180 (36.3%) |  |
| Almost chose an option, n (%) | 45 (7.5%) | 10 (9.7%) | 35 (7.1%) |  |
| Already made a decision, but is still ready to consider, n (%) | 69 (11.5%) | 17 (16.5%) | 52 (10.5%) |  |
| Has already made up their mind and will probably not change their mind, n (%) | 43 (7.2%) | 8 (7.8%) | 35 (7.1%) |  |
| Median | 3 | 3 | 3 | .01 |
| **FTND-R**, median | 7 | 7 | 7 | .10 |

**Note.** DA = decision aid; FTND-R = Revised Fagerström Test for Nicotine Dependence; ^a^excluding the groups 'non-binary' and 'Prefers not to say'; ^1^selecting multiple products was possible; ^2^all dual users, ^3^percentages refer to e-cigarette users only, ^4^excluding extreme outliers ≥1000 and participants that never attempted to stop smoking before, ^5^at least one, can be multiple; percentages exceeding 100% are due to rounding.
