# Supplementary material for: Effects of an Explicit Value Clarification Method With Computer-Tailored Advice on the Effectiveness of a Web-Based Smoking Cessation Decision Aid: Findings From a Randomized Controlled Trial
Source: J Med Internet Res. 2022 Jul 15;24(7):e34246. doi: 10.2196/34246 (PMC9338418; doi:10.2196/34246)
Supplement: Multimedia Appendix 9 [file jmir_v24i7e34246_app9.docx]

**Multimedia Appendix 9.** Results logistic regression H_1b_ (Worst-case scenario adjusted with FTND-R removed): Smoking cessation after 6 months

| ***Worst-case scenario (adjusted)***; R^2^ = .07 (Hosmer-Lemeshow), .03 (Cox-Snell), .09 (Nagelkerke); x^2^(6) = 15.16, *P* = .02^a^, n = 598 | | | | | |
| --- | --- | --- | --- | --- | --- |
|  |  |  | **95% CI for odds ratio** | | |
|  | **B (SE)** | ***P*** | **Lower** | **Odds Ratio** | **Upper** |
| Intercept | -3.84 (0.79) | < .0001 |  |  |  |
| Group allocation (Intervention) | 0.64 (0.36) | .04* | 0.95 | 1.89 | Inf |
| Age (24-29) | -1.94 (1.08) | .07 | 0.01 | 0.14 | 0.82 |
| Age (30-100) | -0.48 (0.42) | .26 | 0.28 | 0.62 | 1.48 |
| Gender (Men) | 0.59 (0.35) | .09 | 0.91 | 1.80 | 3.58 |
| Education (Medium) | -0.17 (0.55) | .76 | 0.31 | 0.84 | 2.74 |
| Education (High) | 0.21 (0.56) | .71 | 0.44 | 1.23 | 4.04 |
| Stage of decision making | 0.31 (0.12) | .01 | 1.08 | 1.36 | 1.71 |

**Note.** *one-sided, ^a^compared to the crude model excluding the non-binary participant.
